# Changes in Ionic Conductance Signature of Nociceptive Neurons Underlying Fabry Disease Phenotype

**DOI:** 10.3389/fneur.2017.00335

**Published:** 2017-07-14

**Authors:** Barbara Namer, Kirstin Ørstavik, Roland Schmidt, Norbert Mair, Inge Petter Kleggetveit, Maximillian Zeidler, Theresa Martha, Ellen Jorum, Martin Schmelz, Theodora Kalpachidou, Michaela Kress, Michiel Langeslag

**Affiliations:** ^1^Department of Physiology and Pathophysiology, University of Erlangen-Nuremberg, Erlangen, Germany; ^2^Department of Anesthesiology, Heidelberg University, Mannheim, Germany; ^3^Section of Clinical Neurophysiology, Department of Neurology, Oslo University Hospital Rikshospitalet, Oslo, Norway; ^4^Department of Clinical Neurophysiology, Uppsala University Hospital, Uppsala, Sweden; ^5^Department of Physiology and Medical Physics, Division of Physiology, Medical University of Innsbruck, Innsbruck, Austria; ^6^Faculty of Medicine, Institute of Clinical Medicine, University of Oslo, Oslo, Norway

**Keywords:** Fabry disease, lysosomal storage disorder, single fiber recordings, neuronal excitability, electrophysiology, nociception

## Abstract

The first symptom arising in many Fabry patients is neuropathic pain due to changes in small myelinated and unmyelinated fibers in the periphery, which is subsequently followed by a loss of sensory perception. Here we studied changes in the peripheral nervous system of Fabry patients and a Fabry mouse model induced by deletion of α-galactosidase A (Gla^−/0^). The skin innervation of Gla^−/0^ mice resembles that of the human Fabry patients. In Fabry diseased humans and Gla^−/0^ mice, we observed similar sensory abnormalities, which were also observed in nerve fiber recordings in both patients and mice. Electrophysiological recordings of cultured Gla^−/0^ nociceptors revealed that the conductance of voltage-gated Na^+^ and Ca^2+^ currents was decreased in Gla^−/0^ nociceptors, whereas the activation of voltage-gated K^+^ currents was at more depolarized potentials. Conclusively, we have observed that reduced sensory perception due to small-fiber degeneration coincides with altered electrophysiological properties of sensory neurons.

## Introduction

Fabry disease is a rare, life-limiting X-linked monogenetic multi-organ disorder caused by deficiency or loss of α-galactosidase A (α-Gal A) (1). Mutations in the α-Gal A encoding *GLA* gene lead to progressive lysosomal accumulation of globotriaosyl-ceramide-3 (Gb3) and related glycosphingolipids. A transgenic mouse, which lacks functional α-Gal A enzyme, is available and serves as a model for the human Fabry disease (2). Symptoms of human Fabry disease include severe pain episodes starting in childhood, followed by autonomic and sensory impairment, which reflects damage to small fibers of the peripheral and autonomic nervous systems, kidney failure, and cardiological as well as other symptoms (3–8). Neuropathic pain is the first symptom that arises in many patients and is due to changes in small myelinated and unmyelinated fibers in the periphery (9). Later in life, the sensory deficits due to small-fiber neuropathy prevail. In line with human findings, transgenic mice modeling the disease show lipid inclusions not only in heart and kidney but also the nervous system (2) and like in patients, these alterations aggravate with increasing age (10, 11). The causal treatment, enzyme replacement therapy that has become available since 2001, is only partially effective and causes immunological intolerance in up to 50% of classically affected men: the enzyme replacement therapy is only partially effective and the long-term efficacy of this costly treatment has been debated (12, 13). Differential treatment responses may correlate with genetic variations within the *GLA* gene where 637 genetic variants including 410 single nucleotide polymorphisms are known to date.^1^ Morphological studies report a reduction of small nerve fibers and deficits in ion channel immunoreactivity in Fabry disease patients suggesting a major contribution of defective primary afferent neurons to the Fabry disease small-fiber neuropathy phenotype (3–7, 14). Lysosomal accumulation of Gb3 is characteristic for Fabry disease, and although Gb3 has been found to alter ion channel function (15–17), its relevance for the pathogenesis of heart, kidney, and neurological deficits including pain is yet incompletely understood. It is unclear whether enzyme replacement therapy has a beneficial effect on the small-fiber neuropathy, loss of sensory perception, and pain experienced by most of the patients (18, 19). Although lyso-Gb3 accumulates in all tissues, the mechanisms for the development and maintenance of sensory and autonomic deficits associated with Fabry disease are still largely enigmatic. A recent report proposes severe small-fiber morphological deficits in Fabry disease patients (20). Based on these findings, we wanted to assess structural and functional properties of sensory neurons in Fabry disease patients and transgenic Fabry disease mice with a combined functional and morphological approach and quantitative assessment of ion channel mRNA expression. Since the Fabry disease-related sensory perception changes with age, we examined middle aged and elderly male Fabry patients and accordingly elderly mice were used in this study (11). This approach suggests large similarities between Fabry disease mice and men and morphological as well as functional deficits in small-fiber neuron subpopulations. Based on these similarities, signatures of neuron function were discovered that can explain sensory deficits associated with Fabry disease.

## Materials and Methods

### Subjects and Clinical Testing

Six male patients with Fabry disease were included. They were all asked about symptoms with special emphasize on pain. All patients were informed orally and in writing. They all gave their written informed consent. All six male Fabry disease patients (age 40–49 years) received enzyme replacement therapy at the time of recording. None of the patients had any other conditions known to cause small-fiber neuropathy. Four patients reported intense pain attacks during childhood especially related to physical activity or fever that were mainly located in their hands and feet, while two of the patients described atypical pain symptoms more related to joint and muscles and one of these especially related to cold environments. All patients had experienced less severe pain symptoms in later years, but were unsure whether this was related to the medication. None of the patients had findings compatible with a large fiber neuropathy when tested with conventional neurography and EMG. However, two patients had pathological findings in median nerve conduction velocity (CV), indicating a moderate carpal tunnel syndrome. They were both asymptomatic. The study was carried out in the Department of Neurology, Oslo university Hospital, Rikshospitalet and in the Department of Clinical Neurophysiology, University Hospital, Uppsala and performed according to the Helsinki Declaration. This was part of a larger study on pain in patients with neuropathy and was approved by the local ethic committees connected to each hospital.

Fifteen healthy subjects aged 41–67 years were used as controls for the microneurography-experiments. These subjects had no known neurological symptoms or diseases and did not use any medication known to influence the nervous system. The results of these healthy subjects have been published previously (21). Part of the study in healthy subjects was done in the Department of Physiology and Experimental Pathophysiology, University of Erlangen/Nürnberg, Erlangen and was approved by the local ethic committee of the University. All participants gave their written informed consent.

### Neurological Examination

The patients underwent a neurological examination including testing of muscular strength in the upper and lower extremities, tendon-reflexes, and sensibility for painful stimuli with a needle, vibration sense, and joint position sense of the first metacarpo-phalangeal joint. Hypersensitivity to gentle touch was assessed by lightly stroking all four extremities with a brush (Somedic). If this light touch was perceived as unpleasant, it was noted as allodynia. The borders of hyperalgesia to punctate stimuli were determined by an 83.7 mN von Frey filament. If this stimulus was reported as more painful when compared with non-symptomatic skin, it was recorded as punctate hyperalgesia. Nerve CVs, amplitudes, and distal latencies were examined with the use of surface stimulation and recording electrodes. The following nerves were studied (orthodromic stimulation): the compound action potential (AP) of the median and ulnar motor nerve fibers and sensory nerve fibers (second and fifth finger) in one random selected extremity, and at least peroneal and tibial motor nerve fibers and sural nerve fibers in one leg in all patients. Threshold (Tresh) temperatures for sensations of warmth, cold, heat-pain, and cold-pain were determined by computerized equipment and with the method of limits (Thermotest^®^, Somedic AB, Sweden). Thermal Treshs were determined from a baseline temperature of 32°C with a 1°Cs^−1^ rate of change. Ten degree Celsius was set as lower and 50°C as upper cut-off temperatures. Warmth detection threshold (WDT) was defined as the lowest temperature above 32°C perceived as warmth, and heat-pain detection threshold (HPDT) as the lowest temperature perceived as painful. Cold detection threshold (CDT) was defined as the highest temperature below 32°C perceived as cold and cold-pain detection threshold (CPDT) as the highest temperature perceived as painful. WDT and CDT were calculated as the average of five consecutive temperature recordings. HPDT and CPDT were determined as the average of three recordings each performed at 10-s intervals. Thermal Treshs were measured at the thenar eminence of one hand, at one lateral thigh, at a lateral site 10–15 cm below knee level, and on the dorso-lateral aspects of both feet. The results were compared with data from 38 healthy subjects (aged 22–66 years) obtained in the laboratory in Oslo (22). The individual Treshs for the patients were considered pathological if they were >95th percentile (WDT, HPDT) or below the 5th percentile (CDT) of values found in healthy subjects. CPDT-values <20°C were considered normal.

### The “Marking Method”

During iterative intra-coetaneous electrical stimulation at a constant frequency of 0.25 Hz for several minutes the C-fiber responses stabilize at latencies characteristic for each unit. It has been shown that even a single additional spike induced in a C-fiber by a conditioning stimulus produces an increased delay of the subsequent electrically induced spike by about 1 ms (23). This “marking” technique is useful to identify which C-fiber in a multi-unit recording responds to a specific stimulus (23, 24). We used the “marking” technique to determine whether C-fibers were spontaneously active or not and to determine the responsiveness of afferent C-nociceptors to mechanical and heat stimuli or additional electrical pulses. The responsiveness of efferent sympathetic C-fibers was tested with the evocation of sympathetic reflexes.

### Activity-Dependent CV Slowing

After a rest period of at least 2 min, an electrical protocol was performed for each stimulation site at the foot; 20 electrical stimuli were applied intra-cutaneously at 0.125 Hz, immediately followed by a second train of 20 pulses at 0.25 Hz and a third train of 30 pulses at 0.5 Hz. Changes in latency were calculated relative to the initial latency (i.e., that immediately following the 120 s rest period). After this test, the stimulation frequency was set to 0.25 Hz for the rest of the experiments. At the end of some experiments, in which the recording conditions were still optimal, we performed a second stimulation protocol which consisted of a train of pulses at 2 Hz for 3 min (25), which was applied after a new rest period of 2 min. To assess the mechanical responsiveness, we stimulated the skin with a stiff von Frey filament (750 mN) repetitively all over an area around the stimulation site (10 cm in diameter), to include innervation territories of both mechano-responsive (CM) and mechano-insensitive (CMi) C-fibers. With a halogen lamp, feed-back controlled by a thermocouple attached to the skin, we assessed the heat responsiveness by increasing skin temperature from 32 to 50°C at 0.25°Cs^−1^. The subjects were instructed to switch off the heat lamp when the heat became too painful. Sympathetic fibers were identified by their marking response to arousal stimuli, e.g., an unexpected loud noise, mental stress, or during deep breath intake (26, 27). This was performed after the step-wise electrical protocol and the efficacy of these maneuvers was controlled by recording background activity of sympathetic burst discharges.

### Classification of C-Fibers

All C-fibers were classified to their sensory (mechanical) responsiveness and axonal properties (activity-dependent slowing behavior) in sympathetic, CM, CMi fibers as previously described (21). Fibers which did show a response to sympathetic stimulation and no response to mechanical or heat stimulation and which showed the typical pattern of slowing for sympathetic C-fibers in the 3 min 2 Hz electrical stimulation protocol were classified as sympathetic fibers (symp). Fibers which did not respond to mechanical stimulation (750 mN) and showed a characteristic activity-dependent CV slowing to repetitive electrical stimulation of more than 5% were classified as CMi. Fibers, which did respond to mechanical stimulation (750 mN) and showed an activity-dependent CV slowing to repetitive electrical stimulation <5% were classified as CM. Fibers which did not match these classification parameters were defined as “atypical.”

### Fabry Disease Mouse Model

The α-Gal A deficient (Gla^−/0^) mice, which were kindly provided by Dr. A. Kulkarni (National Institute of Health, NIDCR, Bethesda, MD, USA), and Wildtype (Wt) C57Bl/6J were bred and genotyped as described previously (2, 28, 29). All mice were maintained under specific pathogen-free conditions, were housed on a 12 h light/dark cycle and had access to food and water *ad libitum*. Littermates were used in all experiments to control for background effects. For all experiments, a statistically weighted number of animals were used. All procedures were in accordance with ethical guidelines and animal welfare standards according to Austrian law and with permission of the Austrian Bundesministerium für Wissenschaft und Forschung (BMWF, BMWF-66.011/0113-II/3b/2010).

### Immunolabeling of Free Nerve Ending in Glabrous Skin

The glabrous skin of the hind paw was dissected from Wt and Gla^−/0^ mice and fixed for 8 h in Zamboni solution [2% PFA and 1.5% picric acid in phosphate-buffered solution (PBS)], cryoprotected in 20% sucrose in PBS at 4°C for at least 16 h and embedded in Tissue-Tek (Sakura-Finetek). Skin cryosection was cut at 20 µm thickness (Leica CM1950) and mounted on poly-lysine coated slides. Glabrous skin sections were blocked with 10% normal goat serum in PBS containing 0.3% Triton X-100 for 1 h and incubated with primary antibodies against β-III tubulin (R&D Systems) at 4°C for 16 h, washed, and incubated at room temperature with α-mouse Alexa Fluor 594 conjugated antibody (ThermoFisher) for 1 h, which included DAPI to stain the nuclei. Z-sections (every 250 nm) were taken on a Zeiss Aviovert 200 M with a 40× oil objective (NA 1.3). Images were processed using ImageJ (only brightness) and quantification of epidermal sensory innervation density was performed as described previously (30). The labeled nerve fibers in the epidermis of at least five randomly chosen z-stacks of three animals per genotype were counted and the fiber density (no. of fibers/1,000 μm^3^) was calculated.

### Experimental Design of Sensory Phenotyping *In Vivo*

In all behavioral experiments, the experimenter was unaware of the genotype of the mice during the whole experiment length. Thermal nociception of Wt and Gla^−/0^ mice was assessed by the Hargreaves method and hot-plate assay. Mice were habituated for at least 1 h before starting the Hargreaves assay. In short, increasing radiant heat is applied to the surface area of hind paw and the time to paw withdrawal is automatically detected (Ugo basile, Italy). Mice were tested three times a day on three separate days at a weekly interval. Before starting the hot-plate assay, mice were placed on a 30°C heated plate for 5 min. Subsequently, the mice were placed on a heated plate of 50°C and the time till the first jump was recorded. Mice were tested on two separate days and the average latency to the first jump was averaged per mouse.

For mechanical sensitivity testing, mice were placed in isolated plastic chambers with a wire mesh floor and allowed to habituate at least 1 h before starting the experiment. The plantar surface of the paw was stimulated by calibrated von Frey monofilaments with a diameter of 1.1 mm. Filaments were applied in an ascending force order (1.4, 4, 8, 16, 22.6, 32, 45.3, and 64 mN). For each monofilament, the frequency of paw withdrawal of 10 stimuli was determined. The Tresh was taken at a 50% paw withdrawal response. If at the strongest filament (64 mN) the withdrawal response of 50% was not reached, the 50% Tresh was set to 64 mN. The mechanical sensitivity was assessed on three separate days with a 7-day interval and Tresh values were averaged per animal.

### Recordings from *Ex Vivo* Skin–Nerve Preparation

To investigate the properties of the afferent nerve fibers innervating the skin of the mouse dorsal hind paw, we used an *in vitro* skin–nerve preparation (31–33). APs of single C-fibers and A-fibers, isolated from the saphenous nerve of Gla^−/0^ mice and Wt C57BL/6J (>20 weeks), were extracellularly recorded. In brief, the saphenous nerve together with the innervated skin of the hind paw was dissected, the preparation placed, epidermis-side down, in an organ bath chamber, and perfused (ca. 12 ml min^−1^) with an oxygen-saturated (95% O_2_/5% CO_2_) modified synthetic interstitial fluid solution containing (in millimolar); NaCl (108), KCl (3.48), MgSO_4_ (3.5), NaHCO_3_ (26), NaH_2_PO_4_ (1.7), CaCl_2_ (2), Na-gluconate (9.6), glucose (5.5), sucrose (7.6) at a temperature of 31.5 ± 0.8°C, and a pH of 7.4 ± 0.05. The distal end of the saphenous nerve was placed in a separate chamber filled with paraffin oil solution to isolate the nerve from the bath solution. Dissected single nerve fibers were placed on a gold wire recording electrode. Recorded APs form single sensory neurons were filtered (low pass 1 KHz, high pass 100 Hz) and stored on a PC-type computer running the Spike/Spidi software package (34). The receptive field was identified by mechanical probing of the skin with a glass rod and afterward electrically stimulated (up to 100 mV) with square-wave pulses (1 ms, stimulus interval 5 s). CVs of the single nerve fibers were calculated from the latency of the AP electrically evoked at the receptive field and the distance between receptive field and recording electrode. The fibers were classified according to their CV; C-fiber < 1.0 ms^−1^, 1.6 ms^−1^ < Aδ-fiber < 12 ms^−1^; and Aβ-fiber > 16 ms^−1^ (31). The mechanical Tresh of each unit was determined with calibrated von Frey filaments with a uniform tip diameter of 1.1 mm by applying increasing forces from 1 to 256 mN, starting with a filament of 22.6 mN. A standardized heat stimulus increased the temperature linearly from 31 to 50°C within 20 s measured at the intracutaneous side. The cold stimulus decreased the temperature from 31 to 3°C within 4 s and held this temperature for 20 s. Fibers were considered responsive if five or more APs were evoked during the stimulus. The Tresh was defined as the force or temperature that elicited the third spike of the response. The isolated fibers were considered spontaneously active when fibers generated four or more APs per minute before mechanical and heat stimuli were applied.

### Neuron Culture

Lumbar dorsal root ganglia (DRG) was harvested from adult mice (age >18 weeks) as previously published (2, 29). After removal of the connective tissue from the ganglia, they were incubated two times in Liberase (Roche, 9 mg 100 ml^−l^ DMEM) for 30 min. After washing with phosphate-buffered saline (PBS), the tissue was incubated with Trypsin-EDTA (0.05%, Gibco, Life Technologies) for 15 min and subsequently washed with TNB medium (Biochrom, Merck Millipore) supplemented with L-glutamin (0.2 mM), Penicillin and Streptomycin (200 U ml^−l^, Gibco, Life Technologies), and Protein-Lipid Complex (Biochrom, Merck Millipore). The DRGs were dissociated with a fire-polished Pasteur pipette and centrifuged through a 3.5% BSA gradient (Sigma) to eliminate debris and non-neuronal cells. The pelleted sensory neurons were resuspended and were plated on coverslips coated with poly-L-lysine/laminin-1 (Sigma), and cultivated in supplemented TNB containing mNGF 2.5 s (Alomone Labs, 25 ng ml^−l^ TNB medium) at 37°C and 5% CO2 in a humidified incubator for 16–24 h.

### Immunolabeling of Cultured DRG Neurons

For immunofluorescence staining of DRG neurons, the DRG neurons were isolated as described above. The neurons were seeded on a laminin coated coverslips and cultured for 24 h at 37°C in a 5% CO_2_, humidified incubator. After 24 h, the cells were fixed with 4% paraformaldehyde. Subsequently, the fixed neurons were stained against neurons specific β-III tubulin and visualized by an α-mouse Alexa Fluor 594 conjugated antibody (ThermoFisher). Images were taken on a Zeiss Axiovert 200 M microscope using a 40× oil objective (NA 1.3) with a scanning stage (ASI M2000). Per slide 20 images were taken and automatically analyzed using a CellProfiler^2^ routine. For each genotype, three independent cultures were analyzed.

### Single Cell Electrophysiology

Cultured sensory neurons were used for electrophysiological experiments 16–24 h after seeding. Glass coverslips were mounted in a recording chamber and placed on a Zeiss Axiovert 200 microscope. All measurements were recorded with an EPC 10 and the Patchmaster v2.73 software (HEKA) at room temperature.

From isolated sensory neurons, cellular voltage recordings were performed in whole-cell current-clamp configuration of the patch clamp technique. The DRG neurons were kept in extracellular solution (ECS) containing (in millimolar); NaCl (150), KCl (5), CaCl_2_ (2), MgCl_2_ (1), HEPES (10), Glucose (10), and the pH was set to 7.3 with NaOH. Borosilicate glass pipettes (Science Products) were pulled with a horizontal puller (P-97, Sutter Instruments Company) and filled with an intracellular solution (ICS) composed of (in millimolar); K-Gluconate (98), KCl (50), CaCl_2_ (0.5), MgCl_2_ (2), EGTA (5), HEPES (10), MgATP (2), NaGTP (0.2), and pH adjusted to 7.3 with KOH. The recorded neurons were held at 0 pA. The minimal current to evoke a single AP within 50 ms (*I*_AP_) for each neuron was obtained by 5 pA increasing depolarizing pulses and amplitude of the 20 s depolarizing pulse was set to 2× *I*_AP_ for the according neuron.

A seven-barrel system with common outlet was used for heat stimulation of single neurons (35). Heat-activated inward currents (*I*_Heat_) were elicited by applying ramp-shaped heat stimuli at 60 s intervals (linear temperature increase from room temperature to 50°C within 5 s). To determine the temperature Tresh of *I*_Heat_, the data were normalized at 25°C and displayed in an Arrhenius plot, where the natural logarithm of *I*_Heat_ was plotted against 1/T (in Kelvin). The Tresh temperature was taken at the intersection of the two linear phases.

The Na^+^ currents were recorded in the whole-cell voltage-clamp as previously published (28). The neurons were kept in an ECS containing (in millimolar); NaCl (90), CsMeS (50), TEACl (10), CaCl_2_ (2), MgCl_2_ (1), HEPES (10), and Glucose (10) and the patch pipettes were filled with ICS composed of (in millimolar); NaCl (10), CsMeS (138), CaCl_2_ (0.1), MgCl_2_ (2), EGTA (1), MgATP (2), and HEPES (10). The recorded sensory neurons were kept at −80 mV holding potential and currents were sampled at 50 kHz and filtered at 2.9 kHz. Currents were triggered by a voltage step protocol (from −120 to +80 mV in 5 mV steps for 50 ms), which was preceded by a −120 mV hyperpolarizing pulse of 250 ms. Tetrodotoxin (TTX, Sigma-Aldrich) was added by bath application the maximal inhibition was monitored in voltage-clamp configuration by applying a depolarizing block pulse protocol to −10 mV for 25 ms from the holding potential.

Extracellular solution for K^+^ current recordings was composed of (in millimolar); NMDG (145), KCl (5), MgCl_2_ (1), HEPES (10), and Glucose (10). The pH was set at 7.3 with HCl. The ICS was as follows (in millimolar); KCl (148), MgCl_2_ (2), CaCl_2_ (0.5), EGTA (5), MgATP (2), NaGTP (0.2), and HEPES (10) and pH was set at 7.3 with KOH. The cells were kept at a holding potential of −80 mV. A 1 s hyperpolarizing prepulse of −120 mV was applied before applying 500 ms depolarizing pulses (Δ10 mV) to investigate the voltage-gated K^+^ currents. To isolate the delayed rectifier K^+^ currents from A-type K^+^ currents, we applied a conditioning prepulse of −40 mV for 1 s to inactivate the A-type potassium currents in the subsequent IV protocol. Subtraction of these K^+^ currents from the K^+^ current evoked from the −120 mV prepulse protocols resulted in A-type K^+^ currents.

For recording of Ca^2+^ currents, the ECS was composed of (in millimolar); NMDG (130), CsCl (12), CaCl_2_ (10), MgCl_2_ (1), HEPES (10), and Glucose (10). The pH was set at 7.3 with HCl. The ICS was defined as follows (in millimolar); CsCl (120), TEACl (20), EGTA (10), MgATP (2), HEPES (10), and sucrose (20) and pH was set at 7.3 with CsOH. The cells were kept at a holding potential of −80 mV. Before applying 250 ms depolarizing pulses (Δ10 mV) to investigate the voltage-gated Ca^2+^ currents, a hyperpolarizing pulse of −120 mV for 100 ms was applied.

A leakage subtraction protocol (P/N 5) was applied preceding all current recordings all currents were normalized for the capacitance of the recorded cell (current density in pA pF^−1^).

### Electrophysiological Analysis

The input resistance of Gla^−/0^ and Wt sensory neurons was determined by four-increasing hyperpolarizing current injections (Δ−5 pA from a holding current of 0 pA, 5 kHz). The average resistance was calculated according to Ohm’s law. From the AP evoked by injecting depolarizing current pulses (50 ms, sampled at 20 kHz), the resting membrane potential (*V*_mem_), afterhyperpolarization (AHP) and overshoot (OS) of Gla^−/0^ and Wt DRG neurons were determined. From the first derivative of the evoked APs, the maximal speed of depolarization and the biphasic repolarization were derived. The AP Tresh was determined where in the falling slope of the 1st derivative reversed into a rising slope and the according membrane voltage was deducted (28).

#### Voltage-Gated Na^+^ and K^+^ Currents

Peak inward currents were plotted against voltage and were fitted with a modified Boltzmann equation with one-step activation phase (Eq. 1)

(1) $$I=I_{\text{Leak}}+G*\left(\frac{1}{1+\text{e}\left(\left(V-V_{\text{act}}\right)/S_{\text{act}}\right)}\right)*\left(V-V_{\text{rev}}\right)$$

where includes conductance *G* (nS.pF^−1^), slope *S* (millivolts), half-activation voltage *V*_act_ (millivolts), and the reversal potential (*V*_rev_ in millivolts). For the K^+^ IVs, the reversal potential was set at −86.0 mV according to the Nernst equilibrium.

#### Voltage-Gated Ca^2+^ Currents

The current–voltage relationships were fitted with a Boltzmann derived equation with one (Eq. 2) or two activation components (Eq. 3)

(2) $$I_{{\text{Ca}}^{\text{2+}}}=I_{\text{Leak}}+G*\left(\frac{1}{1+\text{e}\left(\left(V-V_{\text{act}}\right)/S_{\text{act}}\right)}\right)*\left(\frac{1}{1+\text{e}\left(\left(V-V_{\text{Bl}}\right)/S_{\text{Bl}}\right)}\right)*\left(V-V_{\text{rev}}\right)$$

(3) $$I_{{\text{Ca}}^{2+}}=I_{\text{Leak}}+\left(\left(\frac{G1}{1+\text{e}\left(\left(V-V_{\text{act1}}\right)/S_{\text{act}1}\right)}\right)+\left(\frac{G2}{1+\text{e}\left(\left(V-V_{\text{act2}}\right)/S_{\text{act2}}\right)}\right)\right)*\left(\frac{1}{1+\text{e}\left(\left(V-V_{\text{Bl}}\right)/S_{\text{Bl}}\right)}\right)*\left(V-V_{\text{rev}}\right)$$

where includes conductance *G* (nS.pF^−1^), slope *S* (millivolts), half-activation voltage *V*_act_ (millivolts), and the reversal potential (*V*_rev_ in millivolts).

### RNA Extraction and mRNA Quantification

DRG (L3–L5) were processed as described above. The pellet containing the sensory neurons was resuspended in 500 µl peqGOLD TriFast reagent (peqlab) and processed according to manufacturer’s instructions. RNA quality and quantity was assessed using Nanodrop 2000 (Thermo Scientific). Reverse transcription was performed as previously described (28). Upon transcription, the expression of targets genes was analyzed by quantitative-PCR using TaqMan Gene Expression Assays (ThermoFisher Scientific). The assays used were: Mm00658167_m1 [sodium channel, voltage-gated, type III, alpha subunit, *Scn3a* (Nav1.3)], Mm00488110_m1 [sodium channel, voltage-gated, type VIII, alpha subunit, *Scn8a* (Nav1.6)], Mm00450762_s1 [sodium channel, voltage-gated, type IX, alpha subunit, *Scn9a* (Nav1.7)], Mm01246302_m1 (transient receptor potential cation channel, subfamily V, member 1, *Trpv1*), Mm00446968_m1 (hypoxanthine guanine phosphoribosyl transferase, *Hprt*), Mm01352363_m1 (succinate dehydrogenase complex, subunit A, flavoprotein, *Sdha*), and Mm00441941_m1 (transferrin receptor, *Tfrc*). The reactions were loaded on MicroAmp Fast Optical 96-well reaction plates (ThermoFisher Scientific) and placed in the 7,500 Fast RT-PCR system (ThermoFisher Scientific). The PCR cycle protocol used was: 10 min at 95°C, 40 two-step cycles of 15 s at 95°C and 1 min at 60°C. Each sample was run in duplicates and alongside appropriate controls for each assay was used. Baseline was set manually at 0.1 and threshold cycle (CT) was used as a measure of initial RNA input. Relative fold change in gene expression was calculated using the 2-ΔΔCT method. All fold changes were expressed relative to the respective expression in Wt mice. Three reference genes were used, *Hprt, Sdha*, and *Tfrc*. All three reference genes were found to be stably expressed in both groups of animals, as indicated by geNorm, Normfinder, and Bestkeeper software packages.

### Data-Analyses and Statistics

All values are given as mean ± SEM. Statistical analyses were performed with STATISTICA 6.0 (StatSoft, Tulsa, OK, USA) for the human derived data and were analyzed by multi-way ANOVA with LSD *post hoc* testing and Bonferroni correction when necessary or χ^2^ test. All mouse derived data were analyzed using Origin software (Originlab). Statistical analysis was performed by either Mann–Whitney test in case of non-normal distributed data and *t*-test for normal-distributed data (Shapiro–Wilk).

## Results

### Sensory Neuron Morphology in Gla^−/0^ Mice

**Figure 1:**
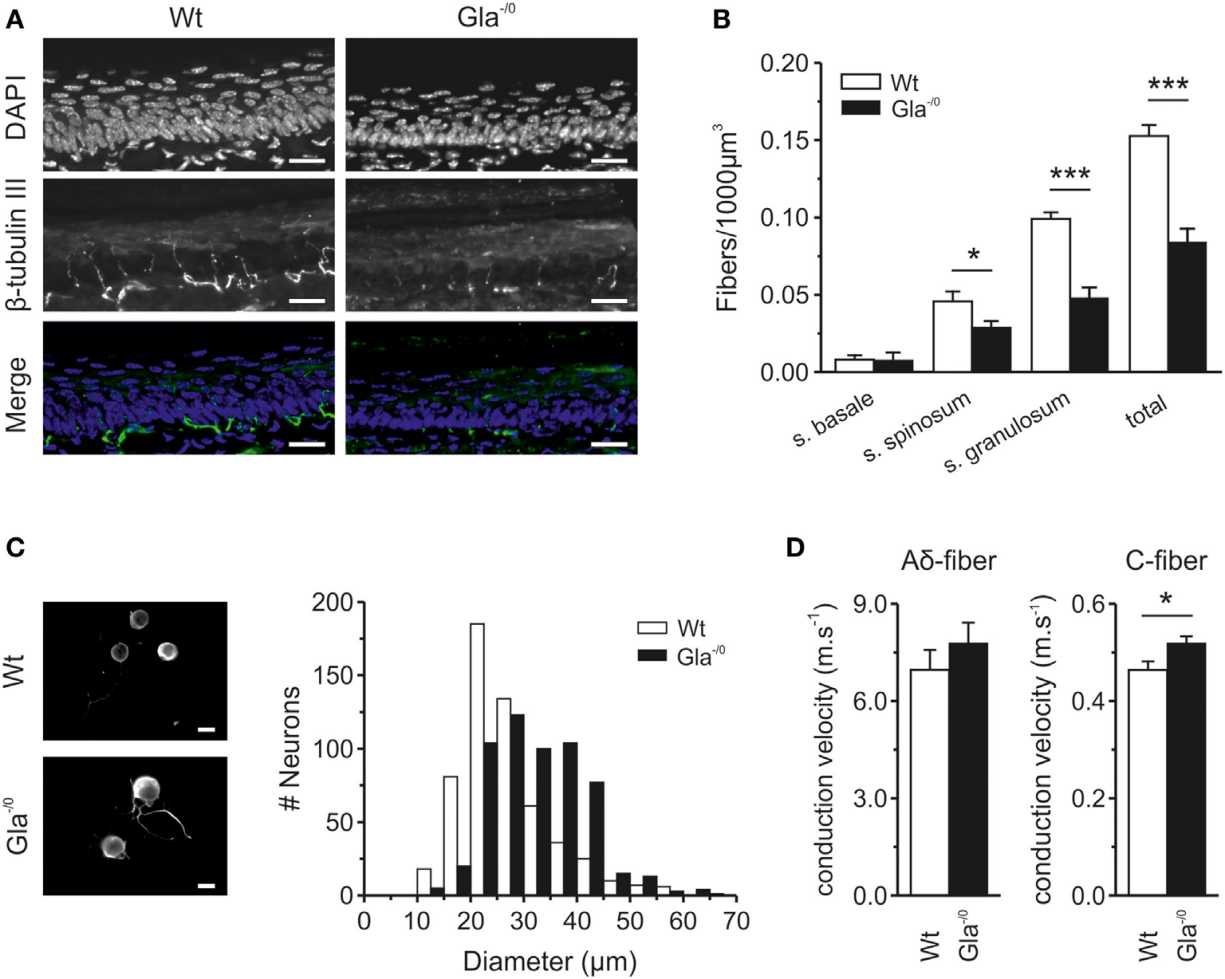
Epidermal nerve innervation of glabrous skin and classification of sensory fibers derived from Wildtype (Wt) and Gla^−/0^ mice. **(A)** Typical immunohistochemical stainings of cryopreserved glabrous skin section (20 µm) from the hind paw of adult Wt and Gla^−/0^ mice, where the nuclei were stained with DAPI (top row) and nerve fibers with anti β-tubulin III (middle row). Images were merged to determine in which layer of the epidermis, based on the morphological shape of the epidermal nuclei, the nerve fibers ended and quantified. **(B)** In all epidermal layers, a reduced number of fibers are detected in Gla^−/0^ glabrous skin. **(C)** The cell body diameter of cultured Gla^−/0^ is increased compare with Wt nociceptors. Representative fluorescent images of cultured Wt (top panel) and Gla^−/0^ nociceptor (lower panel) stained against β-III tubulin showed increased cell bodies of Gla^−/0^ noceptors (scale bar 20 µm). The cell body diameter distribution profile (10 µm bins) of Gla^−/0^ (*n* = 568) and Wt (*n* = 564) nociceptors showed a right shift of the Gla^−/0^ nociceptor cell diameter compared with Wt (χ^2^ test, *p* < 0.001). **(D)** Recorded conduction velocities (CVs) of nociceptive fibers from Wt and Gla^−/0^ mice. The CV of C-fibers of Gla^−/0^ is significantly increased compared Wt C-fibers (right panel), whereas the CV of Aδ-fibers is unchanged (left panel).

Fabry disease patients regularly suffer from small-fiber neuropathy which is associated with neuropathic pain and severe loss of intraepidermal innervation, in particular the thinly myelinated Aδ and unmyelinated C-fibers (6). To explore to which extent the Gla^−/0^ mice, lacking functional α-Gal A, share similarities with Fabry disease patients, the innervation of hindpaw glabrous skin of adult Gla^−/0^ and Wt mice was examined by indirect immune fluorescence microscopy. The innervation depth of the fibers was assessed by the nuclear morphology of the different epidermal layers (s. basale, s. spinosum, and s. granulosum). While the thickness of all skin layers (without s. corneum) was similar in both Gla^−/0^ and Wt mice (Gla^−/0^; 91.87 ± 2.28 µm and Wt; 88.20 ± 2.27 µm, *t*-test, *p* = 0.28), the global density of epidermal innervation of glabrous skin of Gla^−/0^ mice was prominently reduced to approximately 50% of Wt (Figure 1A) with a marked reduction in fibers penetrating the epidermal stratum spinosum and granulosum. However, the number of fibers terminating in s. basale was similar in Wt and Gla^−/0^ mice (Figure 1B). Thus, the changes in fiber morphology in the Fabry disease mouse model closely resembled the pathological cutaneous innervation patterns found previously in Fabry disease patients (20). Such alterations may be due to deficits in neurite outgrowth (36). To test this hypothesis, Gla^−/0^ and Wt sensory neurons were cultured for 24 h and cell body diameters were quantified. While no pronounced deficits in neurite outgrowth were observed, a striking difference in neuronal soma size was discovered. We hardly observed any small sized Gla^−/0^ nociceptors (<20 µm) but a significantly increased number of nociceptors with diameters >30 µm compared with cultured Wt neurons (χ^2^ test, *p* < 0.001, Figure 1C). The average soma diameter of cultured Gla^−/0^ nociceptors (32.38 ± 0.37 µm, *n* = 568) was significantly larger when compared with cultured Wt sensory neurons (26.90 ± 0.36 µm, *n* = 564, MWU, *p* < 0.001).

### C-Fibers of Gla^−/0^ Mice Display Higher Conductance Velocities

To explore functional properties of primary afferents in the Fabry disease mouse model, we performed single fiber recordings from unmyelinated and thin myelinated fibers (C- and Aδ-fibers) in an *in vitro* skin–nerve preparation. All recorded fibers were subjected to electrical, mechanical, and thermal stimuli for fiber classification. We observed that the CV of adult Gla^−/0^ Aδ fibers (7.77 ± 0.65 ms^−1^, *n* = 15) and Wt Aδ fibers (6.96 ± 0.62 ms^−1^, *n* = 18) were similar (Figure 1D, left panel). However, the CV of the Gla^−/0^ nociceptive C-fibers was significantly faster (0.52 ± 0.04 ms^−1^, *n* = 33) than that of Wt C-fibers (0.46 ± 0.04 ms^−1^, *n* = 36, Figure 1D, right panel). This difference was particularly evident in heat-responsive C-fibers (Gla^−/0^; 0.50 ± 0.02 ms^−1^, *n* = 25 vs. Wt; 0.45 ± 0.02 ms^−1^, *n* = 31, MWU, *p* = 0.020). There was no difference between the CV of heat-insensitive C-fibers of Gla^−/0^ and Wt mice (Gla^−/0^; 0.55 ± 0.05 ms^−1^, *n* = 5 and Wt; 0.57 ± 0.03 ms^−1^, *n* = 8, *t*-test, *p* = 0.701).

### Higher CV in Human Fabry Disease Sensory Fibers

**Figure 2:**
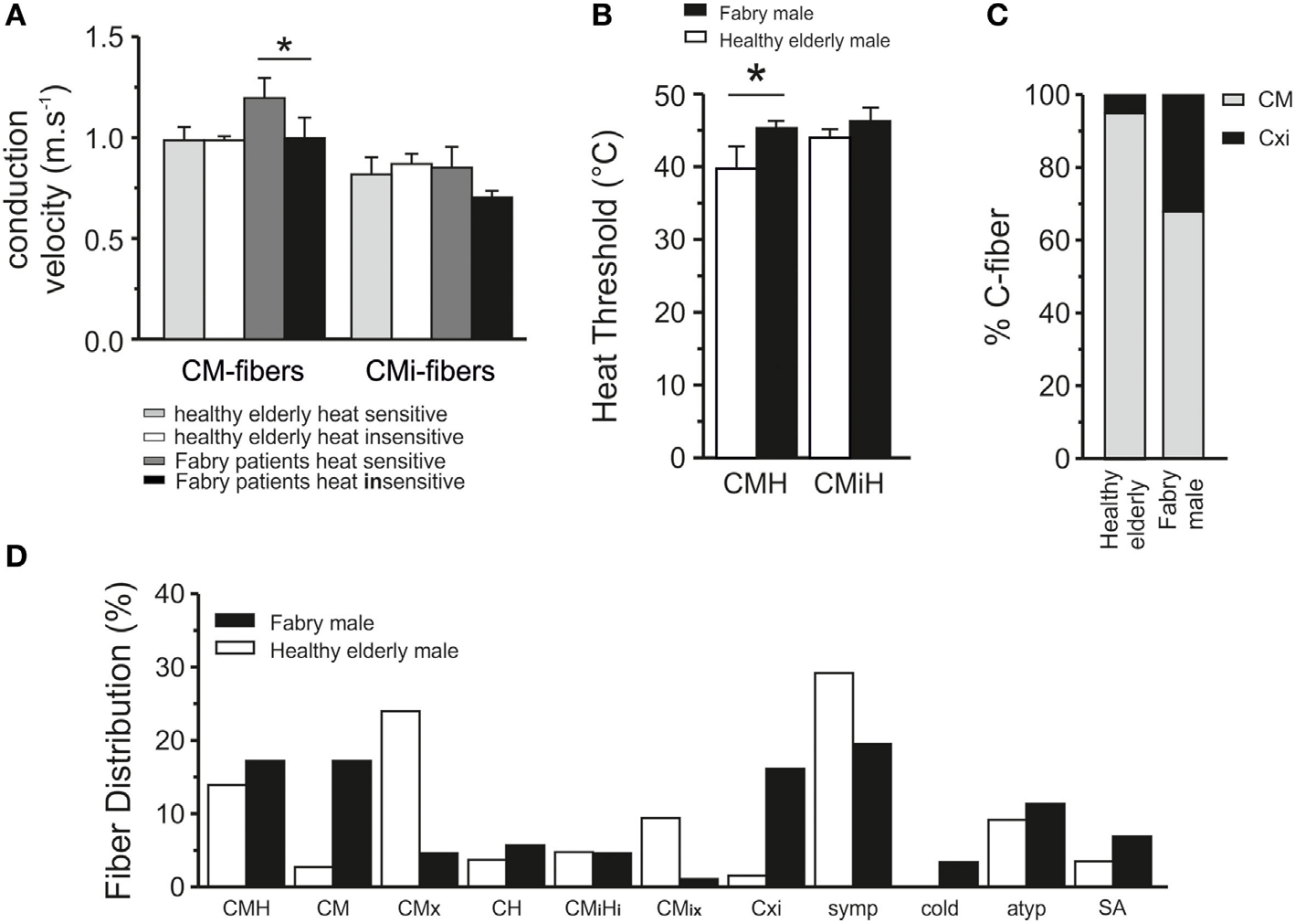
Properties of nociceptive fibers in Fabry disease patients. **(A)** Conduction velocity (CV) in afferent C-fibers with and without heat response in patients with Fabry disease and healthy elderly controls. The mechano-responsive (CM) C-fibers (left side) with heat responses from patients with Fabry disease had higher CVs than those without heat responses (*p* < 0.05; ANOVA, *post hoc* LSD). No significant differences were found in CVs of mechano-insensitive (CMi) C-fibers. **(B)** Heat thresholds in CM C-fibers were significantly higher in patients with Fabry disease than in healthy controls (*p* < 0.05; ANOVA, *post hoc* LSD). The thresholds of CM in patients with Fabry disease were more in the range of CMi C-fibers, which have physiologically higher heat threshsolds. **(C)** In patients with Fabry disease significantly more often C-fibers are found (*p* < 0.05, χ^2^ test), which have biophysical properties of mechano- and heat-responsive C-fibers (CMH) fibers, but show no mechanical response (CXi) than in healthy subjects. **(D)** Proportions of C-fiber subclasses in human patients with Fabry disease and elderly healthy controls. In patients with Fabry disease more afferent C-fibers without heat responsiveness and more fibers without heat and mechanical responsiveness were observed than in healthy controls.

To explore the functional deficits of Fabry disease patients, we performed for the first time microneurographic recordings from primary afferent nerve fibers of human Fabry patients, as previously published (23, 37). A total of 94 C-fibers were recorded and analyzed in Fabry disease patients. Control fibers (230) from healthy subjects were previously published (21). In male Fabry disease patients, mechano- and heat-responsive C-fibers (CMH) fibers exhibited higher CVs in comparison with elderly healthy controls (Figure 2A). In male Fabry patients, the CM C-fibers with a heat response conducted significantly faster than those without heat responsiveness (CM 1.0 ± 0.1 ms^−1^, CMH 1.2 ± 0.1 ms^−1^, *p* = 0.002; ANOVA LSD *post hoc* test, Figure 2A). There was no difference in CV between CM and CMH of healthy elderly subjects. CVs of CMi fibers with and without heat response (CH and CMiHi) did not differ between Fabry patients and healthy elderly (Fabry patients; CH 0.85 ± 0.1 ms^−1^; CMiHi 0.7 ± 0.03 ms^−1^ healthy elderly; CH 0.87 ± 0.1 ms^−1^; CMiHi 0.81 ± 0.1 ms^−1^).

### Alteration of Sensory Fiber Subpopulations, Mechanosensation, and Heat Sensitivity in Fabry Disease Patients

In Fabry disease patients, 34 of the classified fibers (39%) were characterized as CM C-fibers. From 30 tested CM fibers, 15 fibers responded to heat stimulation at the receptive field and were classified as CMH. Ten fibers were classified as CMi and 5 of 9 tested fibers were insensitive to mechanical stimulation but heat responsive (CMiH). In addition, 10 (11%) atypical fibers were identified differing from regular properties of CM and CMi which precluded specific classification. Furthermore, 17 fibers (19.5%) were classified as sympathetic efferents (Figure 2D). In male patients with Fabry disease, we observed that nearly 15% of C-fibers (CM:CXi ratio 1:3) did not react to mechanical stimuli up to 750 mN or heat (CXi) in comparison with <2% unresponsive fibers in healthy elderly subjects (CM:CXi ratio 1:9), indicative of loss of mechano-responsiveness in C-fibers of patients with Fabry disease (Figure 2C, *p* < 0.05, χ^2^ test). In those fibers that responded to mechanical stimulation, the specific mechanical Treshs were not obtained during microneurography recordings.

Psychophysical thermosensitivity was explored in all patients and normal CPDTs were found in all of them. Four of the six male patients had pathological Treshs for cold detection (CDT, Table 1) at their feet, while four had pathological warmth detection threshold (WDT). In all but one of the male patients the Tresh for heat-pain (HPDT) was increased at the feet. These sensory deficits were reflected by corresponding alterations in sensory fiber function as assessed by microneurography. Corresponding heat responsiveness of the CM C-fibers (30 out of 34) was assessed with a ramp-shaped heat stimulus from 34 to 50°C. Only 50% of the tested fibers responded to heat with a mean heat Tresh of 45.2 ± 1.0°C in comparison with 79% in healthy elderly volunteers (21). The heat Treshs of CMH fibers in male Fabry disease patients were significantly higher than in CMH fibers of previously published healthy elderly subjects (21) with 42.7 ± 0.6°C (Figure 2B, *t*-test, *p* < 0.05). In addition, from nine CMi fibers, five fibers responded to the heat stimulus with a mean Tresh of 46.2 ± 1.9°C, which is in the range of healthy elderly volunteers. Heat Treshs of CMiH fibers were similar in male Fabry disease patients and healthy elderly controls (Figure 2B).

**Table 1:** Psychophysical testing of heat sensitivity of recorded fibers from male Fabry patients.

		1	2	3	4	5	6
CDT	Thenar	30.9	30.8	30.9	28.6*	30.8	29.7*
	Thigh	n.d.	27.7	15.6*	27.8	28	29.0
	Leg	n.d.	22.5*	<5*	<5*	29.3	23.2*
	Feet	19.0*	15.7*	23.4*	<5*	26.1	28.7
WDT	Thenar	34.2*	33.6	33.5	35.2*	33.9	35.0*
	Thigh	n.d.	35.7	34.8	35.9	40.7	38.4
	Leg	n.d.	46.6	35.6	45.1	42.3	46.2*
	Feet	39.3	44.0*	41.0	48.1*	47.3*	44.2*
HPDT	Thenar	45.5	44.0	35.4*	39.2	46.4	41.3
	Thigh	n.d.	50.0*	36.6*	38	49.4	42.5
	Leg	n.d.	49.6	38.8	>50*	46.6	49.1
	Feet	48.2*	48.9*	48.1*	>50*	49.6*	47.6

*CDT, cold detection threshold; WDT, warmth detection threshold; HPDT, heat-pain detection threshold; n.d., not determined*.

*Pathological in comparison to the control material the riskshospitalet uses in clinical routine thermal threshold testing.

**Figure 3:**
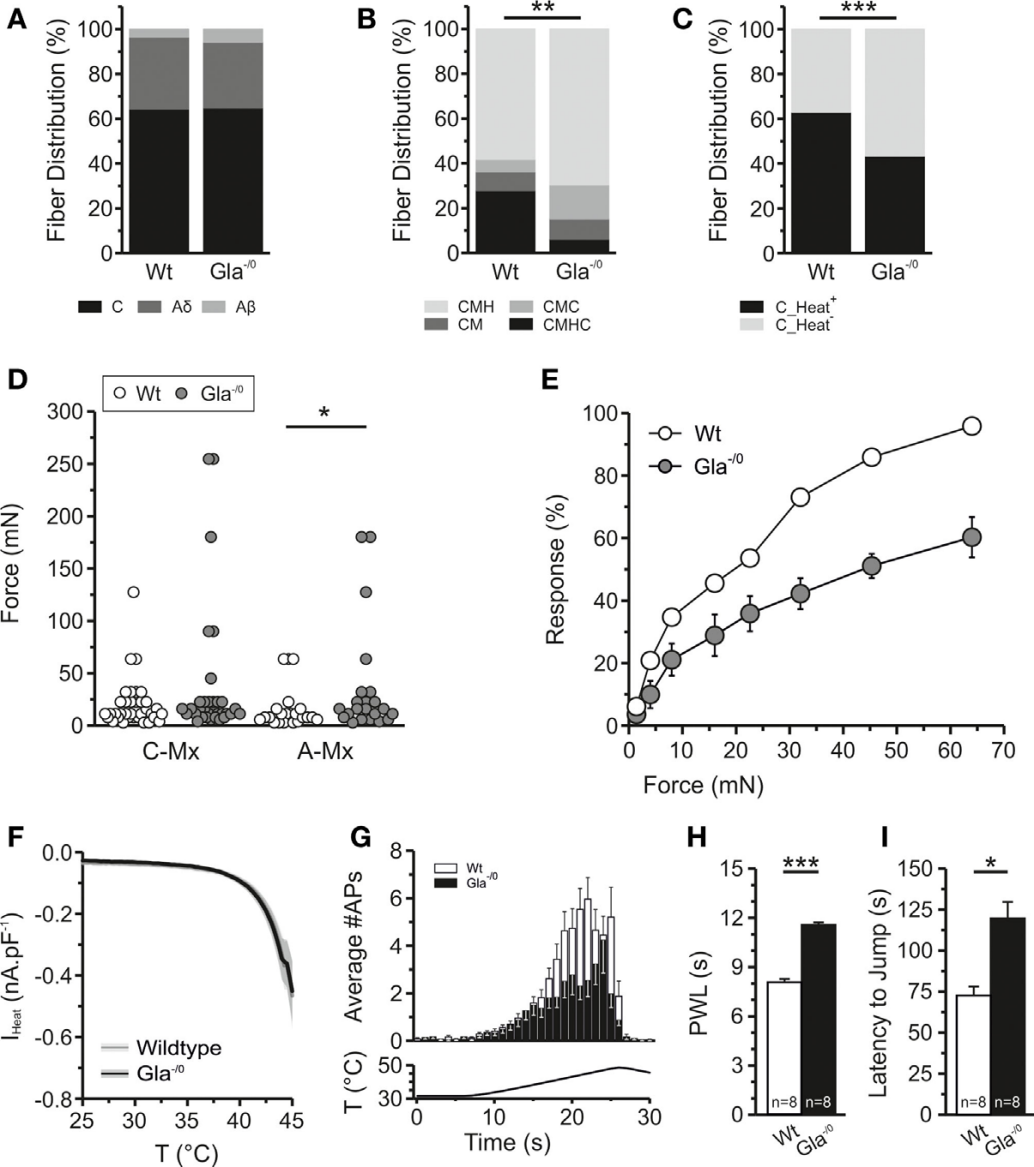
Thermal and mechanical properties of Gla^−/0^ nociceptors. **(A)** The distribution of recorded fibers (Aβ, Aδ, and C-fibers) is not significantly altered in Gla^−/0^ skin–nerve recordings. **(B)** However, within the C-fiber population we observed a significant decrease to the polymodal CMHC fibers and increase of CMH and CMC fibers in Gla^−/0^ samples (***p < 0.01, χ^2^ test). **(C)** Furthermore, there was a significant reduction of heat-responsive fibers (**p < 0.01, χ^2^ test). **(D)** *In vitro* skin–nerve recordings in from 20- to 24-week old Gla^−/0^ and Wildtype (Wt) mice, showed that the free nerve endings of A-fibers in 20–24-week old Gla^−/0^ mice were mechanically hyposensitive (*p < 0.05, MWU). No differences were detected in the mechanical responses of C-fibers. **(E)** The average dose–response curve upon application of von Frey filaments on the hind paw of Wt and Gla^−/0^ mice. The adult Gla^−/0^ mice reduced responses to all filaments when compared with age-matched Wt mice. **(F)** Heat-activated currents derived from cultured Gla^−/0^ nociceptors are identical to the heat-activated currents recorded from Wt nociceptors. **(G)** Free nerve endings in Gla^−/0^ glabrous skin (black bars) generate less action potentials upon a ramp-shaped heat stimulus compared with Wt (white bars). **(H)** Both the Hargreaves test and **(I)**; the hot-plate test (50°C) showed that the adult Gla^−/0^ mice display thermal hyposensitivity compared with Wt mice.

In adult Wt and Gla^−/0^ mice, we found equal percentages of Aβ-, Aδ-, and C-fibers (Figure 3A). In Gla^−/0^, we observed that the percentage of polymodal fibers (CMHC) was significantly reduced, while the fraction of CMH and CMC responsive fibers was increased (Figure 3B). Percentages of cold-responsive C-fibers were similar in Gla^−/0^ and Wt (data not shown), while the fraction of heat-responsive C-fibers was significantly lower in Gla^−/0^ (Figure 3C). This may reflect the increased proportion of heat-insensitive fibers in Fabry disease patients.

### Mechanical Hyposensitivity of Aδ Fibers in Gla^−/0^ Mice

In *ex vivo* skin–nerve preparation in mice, we found no significant difference in mechanical Treshs of C-fibers from Gla^−/0^ vs. Wt mice (Gla^−/0^; 38.87 ± 11.19 mN, *n* = 33 vs. Wt; 20.31 ± 3.83 mN, *n* = 36). However, the mechanical Tresh was significantly increased in the Aδ-fiber population of Gla^−/0^ (Figure 3D; Gla^−/0^; 36.93 ± 11.16 mN, *n* = 22 vs. Wt; 15.83 ± 3.98 mN, *n* = 23, MWU, *p* < 0.05). Sensory phenotyping of Gla^−/0^ mice showed significantly attenuated responses to mechanical von Frey stimulation compared with Wt mice (Figure 3E). Even at the strongest filament tested (64 mN), the responses of the Gla^−/0^ mice were significantly reduced [54.00 ± 1.55% (*n* = 5) vs. 95.83 ± 1.55%, *n* = 6, *t*-test, *p* < 0.001]. Hence, the absolute 50% response rate of the Gla^−/0^ was significantly shifted to higher forces (52.99 ± 2.16 mN, *n* = 5, *t*-test, *p* < 0.001) when compared with Wt mice that reacted at 19.93 ± 0.86 mN (*n* = 6).

### Reduced Thermal Sensitivity of Gla^−/0^ Mice

**Figure 4:**
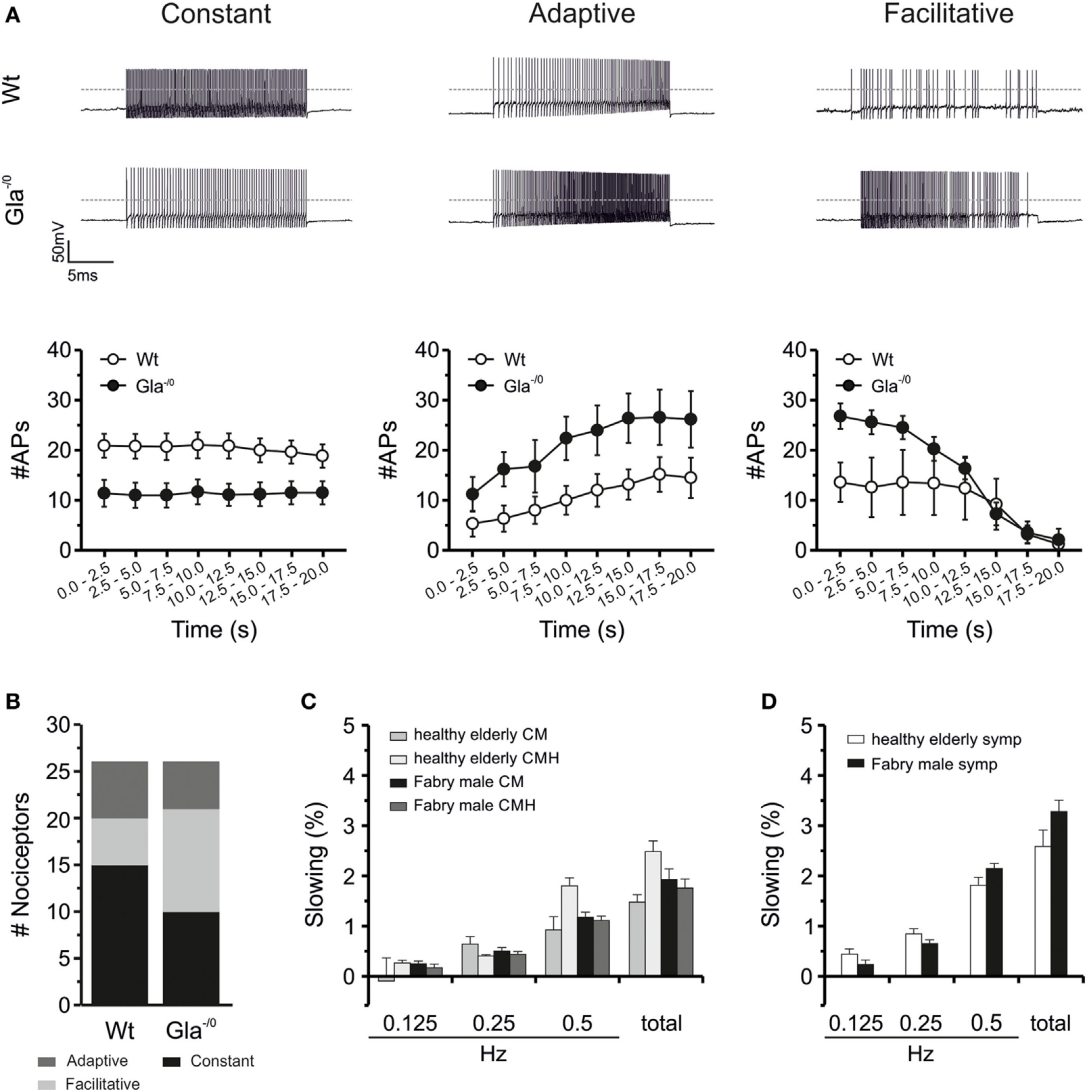
Electrical properties of Gla^−/0^ cultured sensory neurons and activity-dependent slowing of human C-fibers. **(A)** Action potential (AP) generation upon prolonged depolarization significantly changes in adult Gla^−/0^ nociceptors. In matured nociceptors, the constant firing rate in Gla^−/0^ nociceptive neurons is decreased (left panel) and the facilitative and adaptive firing frequency is increased (middle and right panels, respectively). **(B)** Furthermore, nociceptors from Gla^−/0^ mice show an elevated amount of neurons that display a facilitative firing at adult ages and a reduction in the neurons that generate a constant AP firing frequency. **(C)** Activity-dependent conduction velocity slowing (ADS) in C-fibers from patients with M. Fabry and healthy elderly controls showed no significant differences in ADS between heat-responsive and -unresponsive C-fibers. In the left panel, ADS of mechano-responsive C-fibers with (CMH) and without heat response (CM) to electrical stimulation with different frequencies (0.125, 0.25, and 0.5 Hz) and the sum of these ADS steps are depicted. **(D)** ADS of sympathetic C-fibers is shown. No significant differences were found in ADS between patients with M. Fabry and healthy controls.

The human Fabry disease-related changes in Tresh temperatures were only partially consistent with findings from the Fabry disease mouse model. The Tresh temperature of heat-responsive Gla^−/0^ C-fibers (42.13 ± 0.89°C, *n* = 20) was not significantly increased compared with C-fibers recorded from Wt (Figure 4B; 40.55 ± 0.70°C, *n* = 36, *t*-test, *p* = 0.180, Figure S1A in Supplementary Material). To gain more mechanistic insight into the sensory deficits associated with Fabry disease, sensory neurons of Gla^−/0^ and Wt mice were taken into culture and were exposed to ramp-shaped heat stimulus up to 45°C to evoke heat-activated currents (*I*_Heat_). The amplitude of *I*_Heat_ recorded in Gla^−/0^ and Wt sensory neurons were overlapping suggesting no deficit in the transduction mechanism underlying heat nociception (Figure 3F). Likewise, the Tresh temperature of Gla^−/0^ sensory neurons was 42.18 ± 0.86°C (*n* = 21) and similar to the Tresh temperature of *I*_Heat_ recorded from Wt nociceptors (41.11 ± 0.64°C; Figure S1B in Supplementary Material). Accordingly, the mRNA expression of the heat-sensitive ion channel TRPV1 in sensory neurons was similar in both genotypes (Figure S2 in Supplementary Material). However, we observed that Gla^−/0^ heat-responsive fibers generated less APs (32.03 ± 6.54, *n* = 30) upon a ramp-shaped temperature increase in relation to the Wt heat-responsive C-fibers (51.65 ± 5.03, *n* = 49, MWU, *p* < 0.01, Figure 3G). This latter finding is in line with the observation of a sensory deficit when heat-responsiveness was assessed *in vivo*: the paw withdrawal latency in the Hargreaves test was significantly increased in Gla^−/0^ mice (11.54 ± 0.18, *n* = 8) compared with Wt mice (8.04 ± 0.19, *n* = 8, *t*-test, *p* < 0.001, Figure 3H). Furthermore, the adult Gla^−/0^ mice showed delayed nociceptive responses during a 50°C hot-plate assay (Gla^−/0^; 112.90 ± 16.97 s, *n* = 8 and Wt; 67.25 ± 7.36 s, *n* = 8, *t*-test, *p* < 0.05, Figure 3I).

### Reduced Excitability of Cultured DRG Neurons

Since the nociceptive C-fibers of the Fabry disease mouse model showed normal Treshs to thermal and mechanical stimuli but compromised discharge signatures in comparison with Wt C-fibers, we hypothesized that altered excitability of Gla^−/0^ sensory neurons could account for the reduced heat and mechanical responsiveness of Gla^−/0^ mice. Basic membrane biophysical properties such as the input resistance or resting membrane potential of sensory neurons proved to be unaltered in Gla^−/0^ (Table 2). When APs were evoked by depolarizing current injections, the AP characteristics (Tresh, amplitude of OS and AHP) did not differ between both genotypes. However, the minimal current injection needed to evoke a single AP (*I*_AP_) was significantly elevated in Gla^−/0^ (33.85 ± 4.02 vs. 20.19 ± 2.83 pA, *t*-test, *p* < 0.01, Table 2). To assess the discharge characteristics of Gla^−/0^ and Wt nociceptive neurons of the DRG, we injected twice the *I*_AP_ of the recorded neuron for 20 s. The evoked repetitive AP discharge pattern of the neurons could be differentiated in three different types: constant, facilitative [AP frequency (*F*_AP_) increase >1.5 within 20 s] and adaptive (*F*_AP_ decrease <0.67 within 20 s, Figure 4A). Although the population of neurons with adaptive discharge activity was unchanged in Gla^−/0^ (5/26 neurons vs. Wt; 6/26 neurons), the number of neurons that showed the facilitative type was elevated (Gla^−/0^; 11/26 and Wt; 5/26 neurons) while the neurons showing constant firing was lower in Gla^−/0^ (10/26 vs. Wt; 15/26 neurons, χ^2^ test, *p* < 0.05, Figure 4B). When the *F*_AP_ of corresponding groups were compared, it became apparent that Gla^−/0^ nociceptors displayed different kinetics. The constantly discharging Gla^−/0^ neurons showed lower *F*_AP_ during the 20 s depolarization stimulus compared with constant discharge activity Wt neurons. However, both adaptive and facilitative firing Gla^−/0^ neurons had elevated *F*_AP_ when compared with the corresponding Wt neurons. In neurons with adaptive discharge activity, the AP generation was almost completely stalled after 20 s in both genotypes.

**Table 2:** Action potential characteristics of adult Wt and Gla^−/0^ nociceptive neurons.

	Wt (*n* = 27)	Gla^−/0^ (*n* = 26)	*p* (*t*-test)
Input resistance (GΩ)	1.23 ± 0.11	1.20 ± 0.15	n.s.
*I*_AP_ (pA)	20.19 ± 2.83	33.85 ± 4.02	**
RMP (mV)	−48.98 ± 0.74	−49.80 ± 1.01	n.s.
OS (mV)	63.91 ± 2.15	67.27 ± 1.37	n.s.
AHP (mV)	−65.09 ± 0.85	−64.65 ± 0.48	n.s.
Treshold (mV)	−30.80 ± 0.79	−29.94 ± 0.76	n.s.
dV/dt max (mV ms^−1^)	153.40 ± 14.73	185.40 ± 15.02	n.s.
dV/dt min_1_ (mV ms^−1^)	−42.90 ± 3.21	−42.79 ± 2.54	n.s.
dV/dt min_2_ (mV ms^−1^)	−29.86 ± 1.87	−29.59 ± 1.17	n.s.
t1–t2 (ms)	1.36 ± 0.14	1.21 ± 0.08	n.s.
t1–t3 (ms)	4.42 ± 0.40	4.26 ± 0.26	n.s.
t2–t3 (ms)	3.06 ± 0.28	3.05 ± 0.19	n.s.

*I_AP_, minimal current injection (50 ms) to evoke a single action potential; RMP, resting membrane potential; OS, overshoot; AHP, afterhyperpolarization; Wt, wildtype*.

**p < 0.01.

### Signatures of Hyperexcitability in Human Fabry Disease Neurons

Activity-dependent CV slowing in nociceptors was shown to depend on slow inactivation of voltage-gated sodium channels and intracellular sodium accumulation (38–40) and thereby generally accepted as an indirect marker for changes in sodium channel function. According to our classification parameters, the activity-dependent CV slowing in the electrical stimulation protocol with increasing stimulation pulse frequencies (0.125, 0.25, and 0.5 Hz) was significantly larger in CMi units than in CM units. However, there were no differences in slowing between nociceptive fibers from male Fabry disease patients and healthy elderly subjects (Figure 4C), except that in healthy elderly subjects the CMiHi fibers showed significantly more slowing than those in Fabry disease patients (healthy elderly: 8.9 ± 0.3%; Fabry patients: 7.4 ± 2.3%; *p* = 0.007 ANOVA with *post hoc* LSD, data not shown). In the male patients during 2 Hz stimulation for 3 min CMi fibers showed more slowing than CM fibers as expected (37.0 ± 6.8% vs. 15.0 ± 1.2%, *p* < 0.001 ANOVA), which was independent from the heat-responsiveness of these fibers (CM, CMH, CMiHi, and CH). The CXi fibers had a mean slowing of 7 ± 1.2% which is significantly less than the slowing of CM and CMi fiber classes (*p* < 0.001 ANOVA *post hoc*). Sympathetic fibers showed the typical slowing pattern with a plateau and partial reversal of slowing as described before and had a mean maximal slowing of 10.5 ± 0.5% (Figure 4D).

### Alteration of Voltage-Gated Ionic Currents in Gla^−/0^ Sensory Neurons

**Figure 5:**
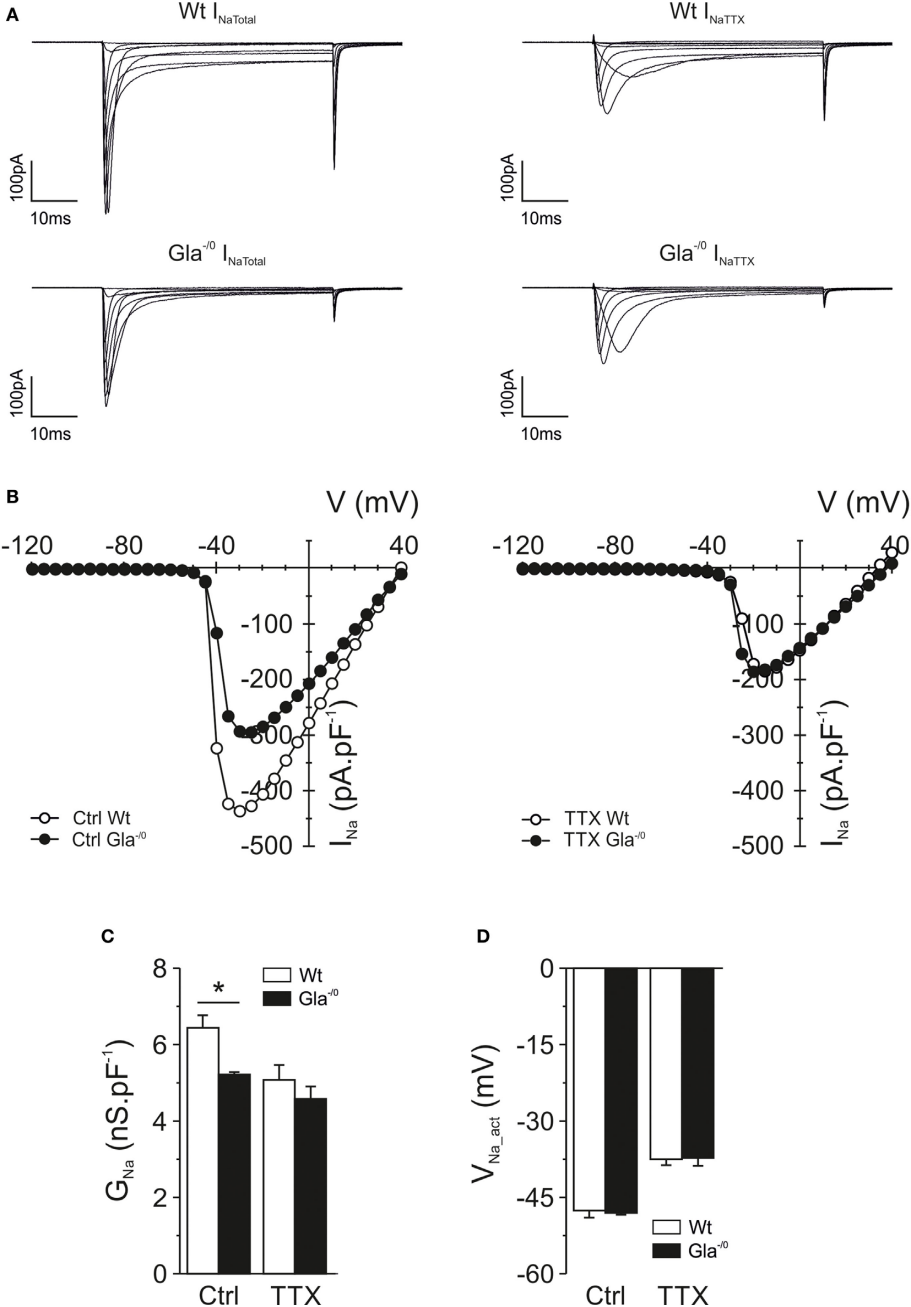
Voltage-gated sodium current recordings in mature Gla^−/0^ and Wildtype (Wt) nociceptors. **(A)** Typical examples of voltage-gated Na^+^ currents from Wt and Gla^−/0^ cultured sensory neurons recorded under normal condition (left panel, *I*_Na Total_) and after application of 250 nm TTX (right panel, *I*_Na TTX_) at depolarizing test pulses from −55 to 15 mV at 10 mV steps. **(B)** IV-curves of voltage-gated Na^+^ currents measured in Wt (white symbols) and Gla^−/0^ (black symbols) nociceptors under control (left panel) and TTX (right panel) conditions. The IV-curves were derived from the recordings shown in **(A)**. **(C)** The Na^+^ conductance of Gla^−/0^ nociceptors was significantly reduced (*p < 0.05, MWU) whereas the **(D)** activation voltage (*V*_Na_act_) was unchanged.

Voltage-gated Na^+^ channels play a pronounced role in AP generation, firing properties, and activity-dependent slowing of nerve fibers. The sensory neurons in the DRG express a multitude of different voltage-gated Na^+^ channels, four of which (Na_V_1.6–1.9) are particularly linked to nociception. Voltage-gated Na^+^ currents were recorded from Gla^−/0^ and Wt nociceptors in whole-cell configuration. The averaged IV-curves obtained from voltage-gated Na^+^ currents under control conditions and in the presence of 250 nM TTX yielded no obvious difference between genotypes (Figure 5A). However, fitting of the individual IV-relationships showed that the conductance (nS pF^−1^) derived from the total Na^+^-IV was significantly reduced in Gla^−/0^ nociceptors compared with the conductance of Wt nociceptors (Figure 5C). No differences were observed in the activation voltage (*V*_Na_act_, Figure 5D) and the slope (data not shown) between both genotypes. Conversely, after application of TTX, the conductance of Na^+^ currents derived from Gla^−/0^ and Wt were comparable as well as the *V*_Na_act_ and the slope (Figures 5A–D and data no shown). Quantification of mRNA expression levels of Na_V_ encoding genes that are characteristic for nociceptive neurons showed differences between Gla^−/0^ and Wt sensory neurons. Altogether, these results suggested that the TTX-sensitive conductance of Gla^−/0^ was smaller than their age-matched Wt controls, independent of the mRNA expression of voltage-gated Na^+^ channels (Figure S2 in Supplementary Material).

**Figure 6:**
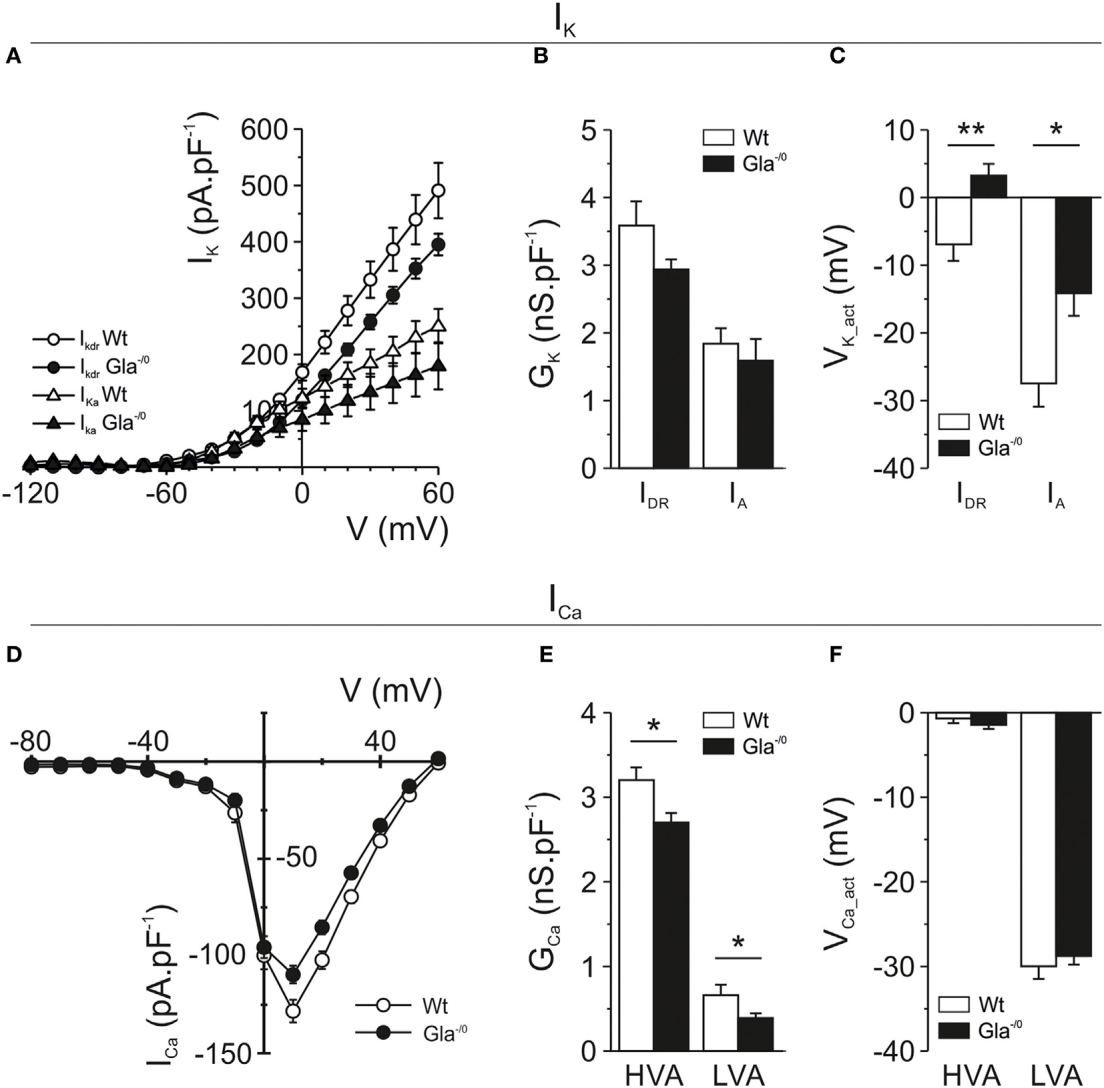
Voltage-gated potassium and calcium currents recorded in mature Gla^−/0^ and Wildtype (Wt) nociceptors. **(A)** IV-plots derived from delayed rectifier K^+^ currents (*I*_DR_, circles) evoked after a 1 s −40 mV prepulse, resulting in inactivation of A-type currents (*I*_A_). Gla^−/0^ dorsal root ganglia (DRG) neurons (black circles) showed decreased *I*_DR_ at depolarized potentials compared with Wt DRG neurons (white circles). In addition, the A-type K^+^ currents of Gla^−/0^ (black triangles) were reduced at depolarized potentials. **(B)** Quantification of the K^+^ conductance revealed no significant differences in the *I*_DR_ and *I*_A_. **(C)** The *V*_act_ of both *I*_DR_ and *I_A_* currents was significantly changed in Gla^−/0^ neurons (black bars). **(D)** IV-plots derived from voltage-gated Ca^2+^ currents showed both Gla^−/0^ (black circles) and Wt (white circles) expressed LVA Ca^2+^ channels, which were activated at more hyperpolarized potentials compared with the HVA channels. The averaged Ca^2+^ IV-plot shows a reduction of HVA Ca^2+^ currents in Gla^−/0^ nociceptors. **(E)** When IV-plots were fitted, the derived conductance of both LVA and HVA Ca^2+^ currents were significantly reduced in Gla^−/0^ sensory neurons (*p < 0.05, MWU). **(F)** The activation voltage of LVA and HVA Ca^2+^ currents were unchanged between Gla^−/0^ and Wt sensory neurons.

The excitability and AP firing behavior of nociceptive neurons does not solely depend on voltage-gated Na^+^ currents, but also on the expression and biophysical behavior of voltage-gated K^+^ channels. Therefore, we also investigated voltage-gated K^+^ currents in Gla^−/0^ nociceptors. The repolarization phase of nociceptive APs consists of two distinct phases, carried by fast activating A-type K^+^ channels and the slowly activated delayed rectifier K^+^ channels. In the Gla^−/0^ DRG neurons, we observed a reduction of delayed rectifier currents in both peak and sustained outward currents (Figure 6A; Figure S3A in Supplementary Material), whereas A-type K^+^ currents did not show a reduced current density (Figure 6B; Figure S3B in Supplementary Material). The activation voltage of both the delayed rectifier (Gla^−/0^; 3.24 ± 1.74 mV vs. Wt; −6.90 ± 2.45 mV, MWU, *p* < 0.01) and A-type K^+^ currents (Gla^−/0^; −14.15 ± 3.34 mV vs. Wt; −27.48 ± 3.42 mV, MWU, *p* < 0.05) were significantly shifted to more depolarized potentials when compared with Wt neurons (Figure 6C).

Finally, voltage-gated Ca^2+^ currents were investigated in sensory neurons which are generally accepted to express low-voltage-activated and high-voltage-activated Ca^2+^ currents. The evoked voltage-gated Ca^2+^ currents in Gla^−/0^ sensory neurons were decreased compared with Wt (Figure 6D). A significantly larger percentage of Gla^−/0^ nociceptors (44.2%) expressed low-voltage-activated Ca^2+^ currents compared with Wt (24.7%, χ^2^ test, *p* < 0.001). However, the conductance of the recorded low-voltage-activated Ca^2+^ currents in Gla^−/0^ nociceptors was significantly decreased (0.39 ± 0.06 vs. 0.66 ± 0.12 nS pF^−1^, *p* < 0.05, MWU, Figure 6E), while the *V*_Ca_act_ (Figure 6F) and the slope (data not shown) remained comparable between both genotypes. The high-voltage-activated Ca^2+^ currents were recorded in all nociceptive neurons. Similar to low-voltage-activated Ca^2+^ currents, the conductance of high-voltage-activated Ca^2+^ currents in Gla^−/0^ nociceptors was decreased (2.70 ± 0.11 nS pF^−1^ vs. Wt; 3.21 ± 0.15 nS pF^−1^, *p* < 0.05, MWU, Figure 6E) and no differences were observed in the *V*_Ca_act_ (Figure 6F) and the slope (data not shown).

## Discussion

The combined approach of the present study supports the Fabry disease mouse as a good model to study Fabry disease-related sensory deficits. Male Fabry disease patients with disease-related small-fiber neuropathy as well as Gla^−/0^ mice, resembling a rodent model of Fabry disease, exhibited similar deficits in nociceptor morphology and function.

The reduced cutaneous fiber density in Gla^−/0^ mice closely resembled the loss of fibers innervating skin in Fabry disease patients (18, 20). Furthermore, this correlates with the increased number of heat- and CMi fibers (CXi) observed in male Fabry disease patients. This subpopulation of fibers was only excited electrically, but not by thermal or mechanical stimuli in microneurography recordings. The increased occurrence of CXi fibers correlated well with the observed gradient in fiber reduction. A possible explanation may be that CXi fibers represents nerve endings, which have degenerated in the uppermost epidermal layers while their more proximal parent axons being still intact and capable of generating and conducting APs, as suggested in patients with diabetes mellitus (22).

In line with these results, heat nociception was significantly reduced in Fabry disease patients and Gla^−/0^ mice. In addition, the psychophysical heat-pain Treshs were increased in Fabry disease patients and were in accordance with the decreased temperature responses of Gla^−/0^ mice in the hot-plate and Hargreaves tests of the present and a previous study (11). In single fiber recordings from both Gla^−/0^ mice and Fabry disease patients, a significantly lower number of heat-responsive C-fibers were detected. The heat-responsive C-fibers in human patients showed elevated heat Treshs, whereas in Gla^−/0^ mice we did not observe any difference in heat activation Treshs of skin-innervating free nerve endings. Differences in heat nociception may be explained by denervation, alterations of heat transduction, e.g., via the heat-sensitive transducer ion channel TRPV1 (41, 42) or by changes of processes involved in AP generation via voltage-gated ion channels (15–17, 42). In cultured Gla^−/0^ nociceptors, the heat-induced current densities and the temperature Treshs were comparable with those of Wt, indicating that heat transduction was unaltered. In line with these results, the TRPV1 mRNA levels in Gla^−/0^ nociceptors appeared to be unchanged compared with Wt. The difference in heat Treshs between human Fabry patients and Gla^−/0^ mice might be caused by a difference in the stage of Fabry disease. The Gla^−/0^ mice used were within their early stage of their lifespan, whereas the human patients were in a later stage of life in which the small-fiber neuropathy might be more advanced. Besides, technical aspects might contribute to the differences in heat Treshs between mice and man: the microneurographic recordings in human Fabry patients are less sensitive for fibers that start firing at a low frequency, whereas the skin–nerve recordings in mice detect every single AP. As shown in the skin–nerve recordings in Gla^−/0^ mice, the temperature Tresh determined by the third AP evoked by the heat stimulus is unchanged, but the firing frequency is reduced. Since spatial and temporal summation is essential for sensation of heat-pain, a reduced firing frequency would result in increased psychophysically assessed heat-pain Treshs as it is present in the here described patients with Fabry disease.

In Fabry disease patients, we observed an increased number of C-fibers that reacted neither to heat nor to mechanical stimulation. Mechanical sensitivity was attenuated in Gla^−/0^ mice as assessed by von Frey testing. Skin–nerve recordings from Gla^−/0^ mice showed no corresponding deficits in mechanical Treshs of Aδ- or C-fibers. Due to the experimental search paradigm of skin–nerve recordings of mice skin, we were unable to record from mechanically insensitive fibers. Together with the loss of skin-innervating fibers in the Gla^−/0^ mice and human Fabry disease patients (18, 20), it is plausible that the loss of mechanical responsiveness was associated with the loss of skin-innervating fibers in our study. This finding contrasts with a previous study reporting decreased mechanical withdrawal Treshs in Gla^−/0^ mice (11). The different observations in the same mouse model further supports previous reports suggesting that mechanical von Frey Treshs may not fully and sufficiently reflect signatures of mechano-nociception in rodent models (43).

Alternatively, age may influence mechanical pain perception as degeneration of small fibers develops with age. Mechanistically, the Fabry disease-related deficits in mechanical and heat nociception may be associated with the degeneration of free nerve endings or changes in AP generation properties, resulting in deficits in pain responses as previously published (2, 44). In both human Fabry patients and Gla^−/0^ mice, we have observed that the CV of sensory fibers is increased compared with healthy elderly and Wt mice. Therefore, we hypothesize that the smallest sensory fibers, which have the lowest CV, might degenerate first as a result of Fabry disease, whereas the larger diameter and/or myelinated fibers are more resistant.

A first indication supporting Fabry disease-related deficits in AP transformation was observed in the heat-responsive fibers in skin–nerve preparations of Gla^−/0^ mice that showed attenuated AP discharge frequency in response to heat stimulation. In cultured Gla^−/0^ nociceptors more subtle changes in excitability could be observed in electrophysiological recordings. The minimal current to evoke a single AP (*I*_AP_) was increased in Gla^−/0^ nociceptors, without any changes observed in the AP parameters. Moreover, we found differential changes in the AP firing frequency upon prolonged depolarization of the nociceptors. In Gla^−/0^ sensory neurons that fired at a constant rate, the AP frequency was reduced, whereas in the adaptive and facilitative firing neurons have an increased firing rate. However, a major challenge lies ahead, since the firing frequency patterns have never been correlated with a specific nociceptor-type or function. It is generally accepted that increased AP firing results in increased pain sensation (45–47), whereas reduction of AP firing, e.g., by analgesics alleviates pain perception (48–50). Since Fabry disease patients suffer from lack of nociceptive responses and on the other hand spontaneous pain attacks and small-fiber neuropathy. Therefore, it would be plausible that the reduced AP frequency in the constantly firing nociceptor population could account for the nociception deficit whereas the adaptive and facilitative firing nociceptors might represent the neurons affected by small-fiber neuropathy and could be involved in the spontaneous pain attacks (51). This fits the observation that the percentage of constantly firing nociceptors was reduced and an increased percentage of facilitative firing nociceptors were recorded in Gla^−/0^ sensory neurons.

Since the Gla^−/0^ mouse model so far appeared well suitable to study Fabry disease-related neuronal alterations, we performed a detailed study of ionic membrane currents which potentially could account for disease-related functional deficits. Voltage-gated Na^+^ channels play a predominant role in pain initiation and propagation. In normal pain perception, the TTX-sensitive Na^+^ channels Na_V_1.7 and Na_V_1.6 have a profound role (52, 53). Mutations in TTX-resistant channels Na_V_1.8 and Na_V_1.9 are associated with painful neuropathy (54, 55). In Gla^−/0^ mice, the conductance of TTX-sensitive currents was reduced, while the conductance of TTX-resistant currents remained unchanged. Although we did not find a decrease of Na_V_ encoding genes, they could be regulated through post-translational modifications (56). Lack of any other major changes in Na_V_ currents correlates to lack of activity-dependent slowing changes in Fabry disease patients as were seen in patients with sodium channel mutations (57–59).

Besides the reduced Na^+^ conductance, we observed reduced conductance for both low- and high-voltage-gated calcium currents which may contribute to the hyposensitivity of Gla^−/0^ mice and Fabry disease patients to noxious stimuli. Low-voltage-activated Ca^2+^ currents carried by T-type voltage-gated Ca^2+^ channels, in sensory neurons take part in regulating neuronal excitability by contributing to the initiation of AP trains (60). Decreased low-voltage-activated Ca^2+^ currents could correspond to reduced pain perception. A decreased conductance of high-voltage-activated Ca^2+^ currents could result in a lower transmission of nociceptive signal into the spinal cord (61) as reported for high-voltage-activated N-type channels (62–64).

On the contrary, we observed an almost twofold increase in the percentage of Gla^−/0^ sensory neurons exhibiting T-type Ca^2+^ currents. This implies that in Gla^−/0^ sensory neurons a small change in membrane potential appears to be sufficient to facilitate the generation of APs upon sub-Tresh stimuli or even spontaneously due to fluctuations of the membrane potential, resulting in neuropathic pain. Nevertheless, we did not observe a corresponding change in *I*_AP_ in Gla^−/0^ sensory neurons that would support this hypothesis. However, T-type channels are also localized at axons, free nerve endings, and neuronal branches in the spinal dorsal horn (65) and activation there would have a greater impact on the resting membrane potential, AP generation, and propagation (66) compared with activation at the soma.

Finally, we observed significant differences in voltage-gated K^+^ currents between Gla^−/0^ and Wt nociceptors which may be of particular importance since voltage-gated K^+^ channels can regulate resting membrane potential, neuronal excitability, and firing frequency (67). In Gla^−/0^ nociceptive neurons, both A-type as well as delayed rectifier K^+^ currents were decreased due to a depolarizing shift in the activation voltage, whereas the conductance of these currents remained unchanged. Although this depolarizing shift does not influence the characteristics of single evoked APs, they could contribute to the increased firing frequency of Gla^−/0^ nociceptors through delayed repolarization during prolonged depolarization. In DRG neurons, A-type K^+^ currents can suppress or delay AP generation and dampen sub-Tresh membrane changes (68). Therefore, the depolarizing shift in the activation voltage of A-type K^+^ currents in Gla^−/0^ nociceptors could prevent attenuation of membrane potential changes that subsequently results in increased excitability of the Gla^−/0^ sensory neuron. However, we cannot exclude the involvement of other K^+^ currents, like M-currents, 2-pore K^+^ currents, and Ca^2+^-activated K^+^ currents that are also well known for their regulation of neuron excitability [for review see Ref. (67, 69)].

Fabry disease-associated pain is generally accepted to be of neuropathic origin, caused by Gb3 accumulation in dorsal root ganglion neurons leading to “spontaneous” pain episodes. Studies have proposed that the spontaneous pain episodes may be caused by hyperexcitability of nociceptive neurons mediated by upregulation of TRPV1 or Na_V_1.8 (41, 42), or increased Ca^2+^ influx by elevated levels of lyso-Gb3 (15, 16). Although our results do not support increased mRNA levels of TRPV1 and Na_V_1.8, these channels may be post-transcriptionally regulated (41, 42). Furthermore, our recordings of voltage-gated Ca^2+^ channels were performed in the absence of exogenous Gb3 and lyso-Gb3. The observed reduction in voltage-gated Ca^2+^ currents in Gla^−/0^ sensory neurons does not omit the potentiating effect of Gb3 or lyso-Gb3 on voltage-gated Ca^2+^ currents (17). Besides, the excitability of the peripheral neurons could also be modulated by changes of descending pathways in the spinal dorsal horn (70) or central nervous system sensitization (71).

Although the mechanism of the change in conductance or activation voltage of voltage-gated ion channels has not been addressed in detail, we observed that cultured Gla^−/0^ sensory neurons exhibited significantly larger cell bodies compared with Wt sensory neurons, which correlates to human Fabry patients where similar observations have been made (72). The cause for this expansion of cell size is currently unknown. However, it may be speculated that the accumulation of α-Gal A substrates in the lysosomes, could lead to increased lysosome numbers and/or size, resulting in an increased cell body volume of Gla^−/0^ sensory neurons in culture. The increased cell body volume could result in increased tension in plasma membrane and thereby affecting the kinetics of voltage-gated ion channels (73, 74).

Fabry disease-associated pain is correlated with increased lyso-Gb3 levels (75) and attenuated with enzyme replacement therapy (76, 77). The symptoms of small-fiber neuropathy improve during enzyme replacement therapy (78). However, an early start of enzyme replacement therapy seems to be critical for alleviating neuropathic pain (17). In some Fabry disease patients, the pain subsides during adulthood even without therapy (79). In the patients that participated in our study, the pain was most severe in childhood and youth and subsided afterward. At the time of the microneurographic study, spontaneous pain was reduced, however, thermal hyposensitivity was more prevalent. It may be speculated whether the enzyme replacement therapy might have been started too late to prevent small-fiber damage and thermal hyposensitivity.

Altogether, the results obtained from Gla^−/0^ mice correlate well with the data obtained from human Fabry disease patients supporting Gla^−/0^ mice as an appropriate model for the assessment of Fabry disease-related pain phenotypes. Electrophysiological recordings from cultured Gla^−/0^ nociceptors showed a reduced excitability, alterations in AP firing behavior, and voltage-dependent ionic currents compared with Wt nociceptors. These observed morphological and functional changes were associated with Fabry disease-related deficits in nociception.

The link to small-fiber neuropathy related pain in Fabry patients to the Fabry disease mouse model might be more complex. While in the early stages of the Fabry disease the peripheral nervous system could be hyperexcitable, in the advanced stages of the disease it might shift to a hypoexcitable state. Furthermore, differential changes within fiber subtypes and/or firing behavior could lead to decreased perception of acute painful stimuli and/or episodes of spontaneous pain. Therefore, one has to be careful to choose, i.e., appropriate age, testing procedures, and experimental read-outs when assessing mechanisms of spontaneous pain and neuropathy.

## Ethics Statement

All human patients were informed orally and in writing and all gave their written consent. The study was carried out in the Department of Neurology, Oslo university Hospital, Rikshospitalet and in the Department of Clinical Neurophysiology, University Hospital, Uppsala and performed according to the Helsinki Declaration. This was part of a larger study on pain in patients with neuropathy and was approved by the local ethic committees connected to each hospital. All animal procedures were in accordance with ethical guidelines and animal welfare standards according to Austrian law and with permission of the Austrian Bundesministerium für Wissenschaft und Forschung (BMWF, BMWF-66.011/0113-II/3b/2010).

## Author Contributions

Conceived and designed the experiments: BN, KØ, MS, EJ, MK, ML. Human data collection: BN, KØ, RS, IK, MS, EJ. Human data analysis: BN, KØ, IK, MS, EJ. Murine data collection: NM, MZ, TM, TK, ML. Murine data analysis: NM, MZ, TM, TK, MK, ML. Wrote the manuscript: BN, MK, ML. All authors read and approved the final manuscript.

## Conflict of Interest Statement

The authors declare that the research was conducted in the absence of any commercial or financial relationships that could be construed as a potential conflict of interest.

## Footnotes

^1^http://fabry-database.org.

^2^http://cellprofiler.org.
