# Supplementary material for: Changes in Ionic Conductance Signature of Nociceptive Neurons Underlying Fabry Disease Phenotype
Source: Front Neurol. 2017 Jul 14;8:335. doi: 10.3389/fneur.2017.00335 (PMC5510289; doi:10.3389/fneur.2017.00335)
Supplement: Supplementary file 1 [file Data_Sheet_1.DOCX]

Supplementary Material

Fabry disease related changes in peripheral sensory neuron function in men and mice

Barbara Namer, Kirstin Ørstavik, Roland Schmidt, Norbert Mair, Inge Petter Kleggetveit, Maximillian Zeidler, Theresa Martha, Ellen Jorum; Martin Schmelz, Theodora Kalpachidou, Michaela Kress, Michiel Langeslag^*^

*** Correspondence:** Michiel Langeslag: michiel.langeslag@i-med.ac.at

## Supplementary Figures


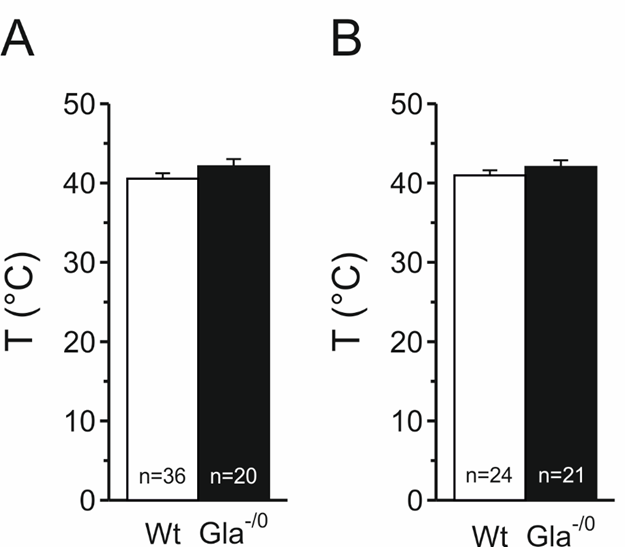


**Supplementary Figure 1.** P**erception of heat by sensory neurons and fibers are unaltered in Gla-/0 mice. (**A) Temperature threshold of sensory C-fibers from Gla^-/0^ mice derived from single fiber recordings is similar to the temperature threshold of wildtype C-fibers innervating the glabrous skin. (B) Cultured sensory neurons from wildtype and Gla^-/0^ elicit heat-activated currents (I_Heat_) at the same temperature when stimulated with a ramp shaped temperature increase.

**
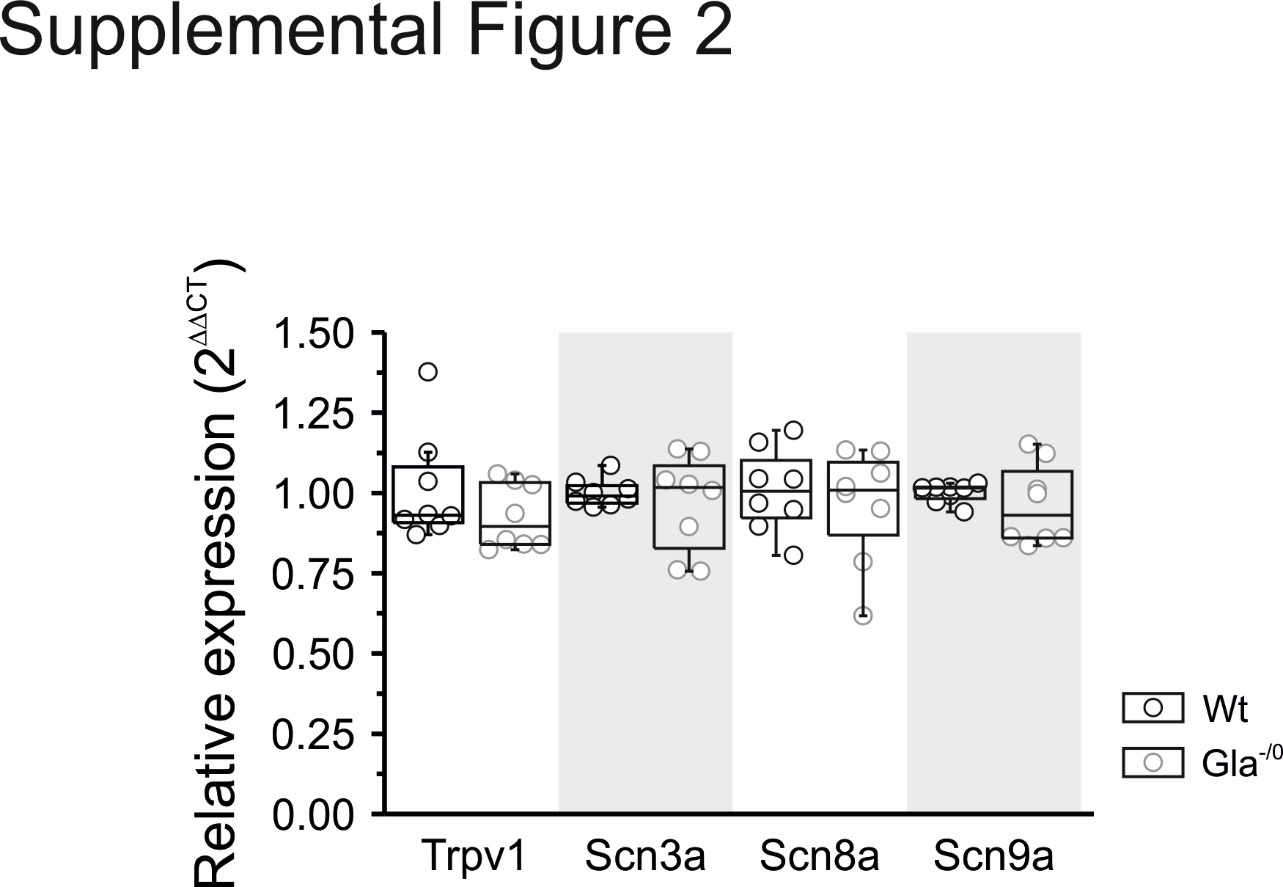
**

**Supplemental Figure 2. mRNA expression of ion channels involved in nociception are unchanged in Gla^-/0^ sensory neurons.** The relative mRNA expression of the heat-responsive TRPV1 channel is unchanged in Gla^-/0^ sensory neurons. Furthermore, expression of voltage-gated Na^+^ channels that are involved in nociception (*Scn3a*, *Scna8a* and *Scn9a*) is comparable between
Gla^-/0^ and wildtype derived sensory neurons.

**
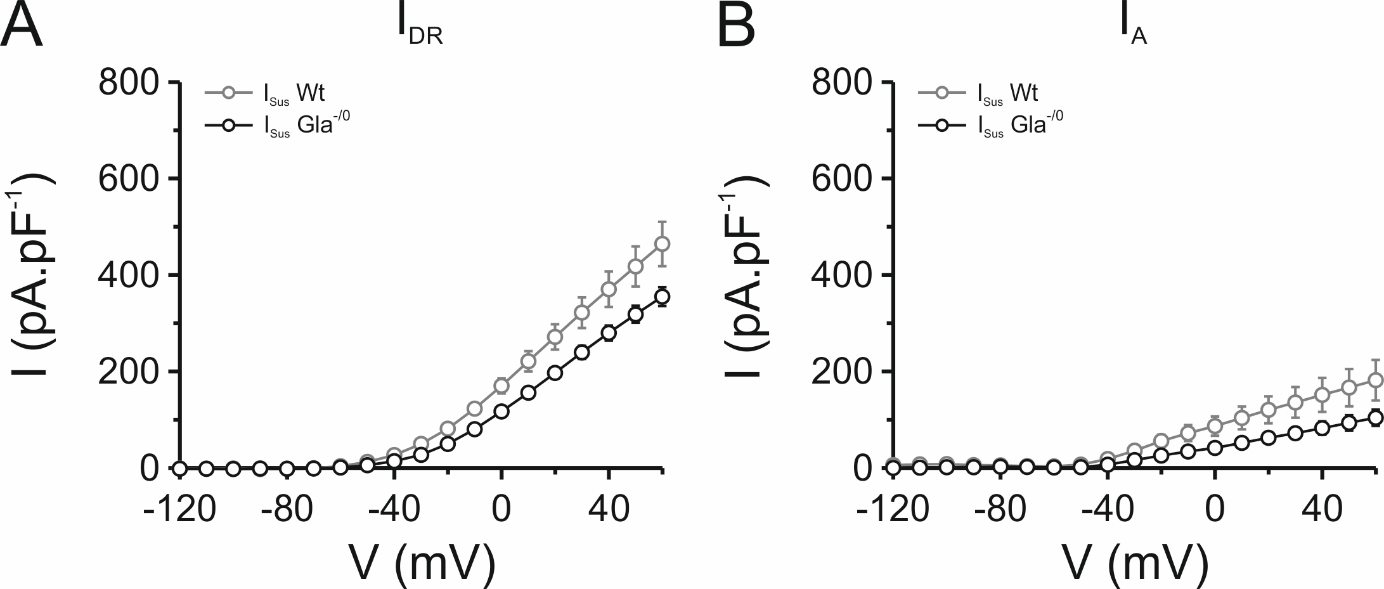
**

**Supplemental Figure 3. Sustained outward K^+^ currents are reduced in Gla^-/0^ nociceptors.**

(A) The current-voltage relationship showed a reduction of the sustained outward current of delayed rectifier K^+^ currents (I_DR_) in Gla^-/0^ sensory neurons (black open circles) compared to the sustained I_DR_ recorded from wildtype sensory neurons (grey open circles). (B) Similar to the sustained I_DR_, the sustained K^+^ current of A-type K^+^ currents (I_A_) is decreased in Gla-/0 sensory neurons (black open circles) in comparison with wildtype sustained I_A_ (grey open circles)
